# Acceptability, Usability, and Insights Into Cybersickness Levels of a Novel Virtual Reality Environment for the Evaluation of Depressive Symptoms: Exploratory Observational Study

**DOI:** 10.2196/68132

**Published:** 2025-04-16

**Authors:** Sara Sutori, Emma Therése Eliasson, Francesca Mura, Victor Ortiz, Vincenzo Catrambonephd, Gergö Hadlaczky, Ivo Todorov, Antonio Luca Alfeo, Valentina Cardi, Mario G C A Cimino, Giovanna Mioni, Mariano Alcañiz Raya, Gaetano Valenza, Vladimir Carli, Claudio Gentili

**Affiliations:** 1 National Centre for Suicide Research and Prevention Department of Learning, Informatics, Management and Ethics Karolinska Institutet Stockholm Sweden; 2 Padova Neuroscience Center University of Padua Padua Italy; 3 Instituto Universitario de Investigación en Tecnología Centrada en el Ser Humano Universitat Politècnica de València Ciudad Politécnica de la Innovación València Spain; 4 Research Center E. Piaggio Department of Information Engineering University of Pisa Pisa Italy; 5 Department of General Psychology University of Padua Padua Italy

**Keywords:** depression, virtual reality, assessment, acceptability, usability, cybersickness

## Abstract

**Background:**

There is a clear need for enhanced mental health assessment, depressive symptom (DS) evaluation being no exception. A promising approach to this aim is using virtual reality (VR), which entails the potential of adding a wider set of assessment domains with enhanced ecological validity. However, whilst several studies have used VR for both diagnostic and treatment purposes, its acceptance, in particular how exposure to virtual environments affects populations with psychiatric conditions remains unknown.

**Objective:**

This study aims to report on the acceptability, usability, and cybersickness levels of a pilot VR environment designed for the purpose of differentiating between individuals with DSs.

**Methods:**

The exploratory study, conducted in Italy, included 50 healthy controls and 50 young adults with mild-to-moderate DSs (without the need for a formal diagnosis). The study used an observational design with approximately 30 minutes of VR exposure followed by a self-report questionnaire battery. The battery included a questionnaire based on the Theoretical Framework of Acceptability, the System Usability Scale as well as the Simulator Sickness Questionnaire.

**Results:**

Results indicate that the majority found VR acceptable for the purposes of mental health screening and treatment. However, for diagnostics, there was a clear preference for VR to be used by mental health professionals as a supplementary tool, as opposed to a stand-alone solution. In practice, following exposure to the pilot VR environment, generally, good levels of acceptability and usability were reported, but areas in need of improvement were identified (such as self-efficacy). Self-reported cybersickness levels were comparable to literature averages but were considerably higher among those with DSs.

**Conclusions:**

These findings raise questions about the potential interplay between underlying somatic symptoms of depression and VR-induced cybersickness and call for more attention from the scientific community both in terms of methodology as well as potential clinical and theoretical implications. Conclusively, user support indicates a potential for VR to aid mental health assessment, but further research is needed to understand how exposure to virtual environments might affect populations with varying severity and other forms of psychiatric symptoms.

**International Registered Report Identifier (IRRID):**

RR2-10.1186/ISRCTN16396369

## Introduction

### Background

In 2019, an estimated 970 million people worldwide, or 12.6% of the global population, were living with a mental disorder, with anxiety (301 million) and depression (280 million) being the most prevalent [1]. These conditions contribute substantially to the global burden of disease, with depression being one of the leading causes of disability worldwide [2]. Despite considerable efforts to address this issue, there has been no substantial reduction in the burden of mental disorders since 1990 [1].

Depressive disorders (hereafter referred to as depression) present significant challenges in research, among other reasons due to the diagnostic uncertainty [3], characterized by both high heterogeneity and low reliability [4]. The persistence of these challenges over different iterations of the *DSM* (*Diagnostic and Statistical Manual of Mental Disorders*) [4] underscores the need to advance clinical practice, which currently predominantly relies on self-report questionnaires and clinical interviews [5].

In response to the challenges mentioned above, suggestions have been made to include a broader range of measures that cover more assessment domains and are not solely dependent on verbal self-report [6,7]. Such measures include speech, text, and facial expression analysis [8]; genomics, transcriptomics, proteomics, metabolomics, and imaging [9]; behavioral and physiological variables collected via wearable sensors [10] or virtual reality (VR) systems [7].

VR systems are particularly relevant to mental health disorders due to immersive, controlled, and customizable environments that can be tailored to individual needs [7]. VR has been used across psychiatric pathologies but reviews consistently report that depression has remained understudied compared to other conditions [7,11,12]. The gap in the literature is particularly evident for studies on assessment, of which we could only find three [13-15]. These are specifically focusing on attentional processes [14], spatial navigation [15], and social interactions [13] in relation to depression. Despite these pioneering studies, the potential of VR to integrate multiple domains of data for enhancing assessment [7] has yet to be explored.

While efforts are being dedicated to improving treatment and the testing of various measures to enhance assessment, including the use of VR, even less attention has been paid to the evaluation of such novel approaches from a user perspective. Research on VR for depression treatment and assessment is limited, with even fewer studies focusing on its acceptability, usability, and tolerability. Beyond diagnostic accuracy, evaluating the acceptability and usability of diagnostic tools is crucial, particularly when involving individuals with mental health conditions, like depression. In such cases, where patients might face additional treatment barriers, including motivational difficulties, stigma, delayed help-seeking [16], or poor treatment adherence [17], acceptability and usability play a crucial role in the successful implementation of new diagnostic and treatment technologies.

Sekhon et al [18] define acceptability as a “multifaceted construct that reflects the extent to which people delivering or receiving a health care intervention consider it to be appropriate, based on anticipated or experienced cognitive and emotional responses to the intervention.” On the other hand, usability has various definitions and approaches [19], but here we will refer to the extent to which the VR system can be used with ease for the purpose of assessing depression symptom severity. Third, tolerability may be defined in different ways, but here will refer to cybersickness severity. Cybersickness describes a visually induced motion sickness-like discomfort during or following exposure to VR. Cybersickness is accompanied by changes in the activity of the central and autonomic nervous systems and results in symptoms like headache, nausea, and eyestrain among others [20].

To the best of our knowledge, only 2 studies using VR have reported on the earlier concepts in relation to depression: one focusing on psychoeducation [21] and another on behavior activation [22]. They both found generally high acceptability and satisfaction levels. Migoya et al [21] also found favorable ratings for ease of use and perceived usefulness, whereas Paul et al [22] reported a decreasing interest in continuing the program after having attended multiple sessions. Paul et al [22] also assessed cybersickness, noting decreasing symptoms as the sessions progressed, but their unconventional scoring method (adding up raw scores as opposed to using weight adjustments) complicates comparisons with studies on healthy controls (HCs) [23] or other psychiatric conditions [24]. To date, no studies have compared cybersickness levels between depressed individuals and HCs.

To tackle the aforementioned challenges, this study introduces a novel approach aimed at enhancing the quality—including the breadth and ecological validity—of depression assessment. A VR system and environment were developed to capture physiological data (heart rate, skin conductance, and eye tracking), behavioral data (curiosity-related behavior and attentional biases), and cognitive data (working memory, processing speed, sustained attention, cognitive flexibility, self-evaluation, and metacognitive sensitivity) hypothesized to be associated with depression [25]. These multidomain data are then analyzed via machine learning models and explainable artificial intelligence to assess depressive symptom (DS) severity. The categorization accuracy of this system is reported on in a dedicated study [25], however, given the innovative nature of this approach and the gap in the literature, it is fundamental to consider the patient perspective as well. Therefore, the objective of this study was to evaluate the acceptability, usability, and cybersickness levels of this pilot virtual environment.

### Aim

The aim of the study was to assess the acceptability, usability, and cybersickness levels of a pilot VR environment designed to aid and enrich the assessment of DSs.

## Methods

### Overview

The study had an exploratory nature and followed an observational design, where all participants completed the same questionnaire batteries before and post VR and followed the same VR exposure protocol.

### Sample

**Figure 1:**
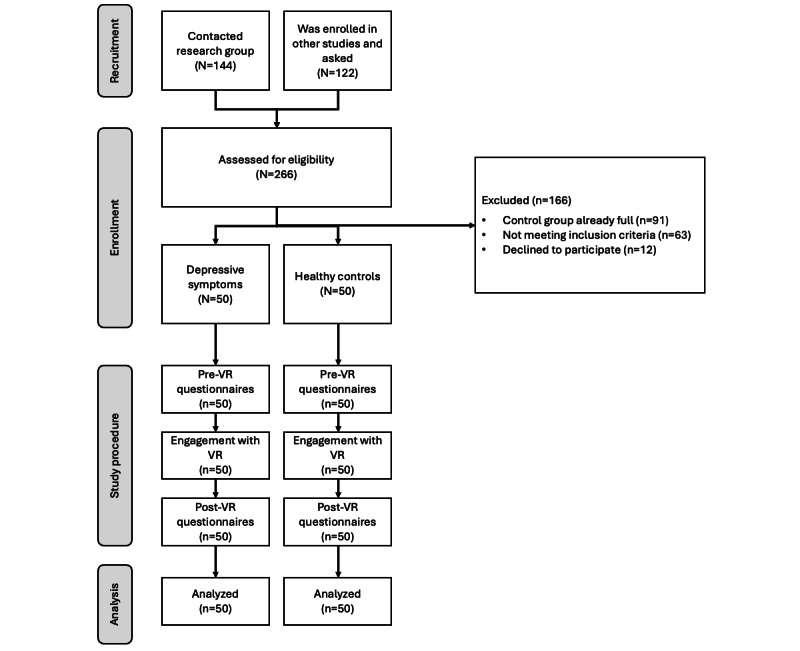
Consort flow diagram of participant involvement. VR: virtual reality.

A total of 266 individuals were screened at the University of Padua, Italy, with continuous enrollment between November 2022 and November 2023 (Figure 1). The screening concerned the inclusion and exclusion criteria detailed later and was done using self-report questionnaires administered in Qualtrics (Qualtrics, Inc). Recruitment was completed when the predefined target sample size of 100 was reached (Figure 1), including 50 participants with DSs and 50 participants classed as HCs. Participants were aged 18-35 years, fulfilled all inclusion criteria, and provided written informed consent. Further demographic and clinical characteristics of the sample are outlined in Table 1.

**Table 1:** Demographic and clinical characteristics of study participants by study groups.

			DS^ab^ (N=50)		HC^c^ (N=50)		Between group difference, *r*_*rb*_^d^
**Sex, n (%)**							
	Male	10 (20)		10 (20)		—^e^	
	Female	40 (80)		40 (80)		—	
Age (years), mean (SD)			23.0 (1.86)		23.3 (1.52)		0.13
Education (years), mean (SD)			16.1 (1.40)		16.6 (0.78)		0.14
**Hand dominance, n (%)**							
	Left	0 (0)		3 (6)		—	
	Right	50 (100)		47 (94)		—	
**Sight correction, n (%)**							
	None	21 (42)		26 (52)		—	
	Lenses	10 (20)		10 (20)		—	
	Glasses	19 (38)		14 (28)		—	
Height (meter), mean (SD)			1.67 (0.07)		1.66 (0.07)		0.07
Weight (kg), mean (SD)			62.7 (10.60)		59.6 (7.85)		0.12
BMI, mean (SD)			22.4 (3.00)		21.5 (2.06)		0.13
Habitual sleep (hours/night), mean (SD)			7.0 (0.98)		7.3 (0.88)		0.20
**Habitual smoker, n (%)**							
	Yes	19 (38)		20 (40)		—	
	No	31 (62)		30 (60)		—	
**Habitual consumption of alcohol, n (%)**							
	Yes	11 (22)		16 (32)		—	
	No	39 (78)		34 (68)		—	
**Habitual user of psychoactive drugs, n (%)**							
	Yes	11 (22)		4 (8)		—	
	No	39 (78)		46 (92)		—	
PHQ-9^f^, mean (SD)			11.3 (1.92)		3.2 (1.48)		0.87^g^
DASS-D^h^, mean (SD)			15.2 (7.40)		3.5 (3.44)		0.77^g^
DASS-A^i^, mean (SD)			9.6 (6.62)		2.4 (2.89)		0.61^g^
DASS-S^j^, mean (SD)			17.8 (6.73)		7.7 (5.08)		0.66^g^
PANAS-Pos^k^ before VR^l^, mean (SD)			10.4 (3.53)		12.2 (2.76)		0.27^m^
PANAS-Pos after VR, mean (SD)			8.9 (4.36)		11.8 (3.61)		0.36^g^
PANAS-Neg^n^ before VR, mean (SD)			3.2 (2.98)		1.9 (2.22)		0.24^o^
PANAS-Neg after VR, mean (SD)			3.2 (3.25)		1.7 (2.24)		0.24^o^

^a^The categorization is based on current depressive symptom severity assessment via the Patient Health Questionnaire-9 (score equal to 9 or above) and not the presence of a clinical diagnosis.

^b^DS: depressive symptom.

^c^HC: healthy control.

^d^Group differences are calculated via 2-tailed Mann Whitney *U* tests and effect sizes reflect rank-biserial (*r_rb_*) correlations.

^e^—: not applicable.

^f^PHQ-9: Patient Health Questionnaire-9.

^g^*P*<.001.

^h^DASS-D: Depression Anxiety Stress Scale-Depression.

^i^DASS-A: Depression Anxiety Stress Scale-Anxiety.

^j^DASS-S: Depression Anxiety Stress Scale-Stress.

^kj^PANAS-Pos: Positive and Negative Affect Schedule – Positive affect

^l^VR: virtual reality.

^m^*P*<.01,

^n^PANAS-Neg: Positive and Negative Affect Schedule – Negative affect

^o^*P*<.05,

### Power Calculation

**Figure 2:**
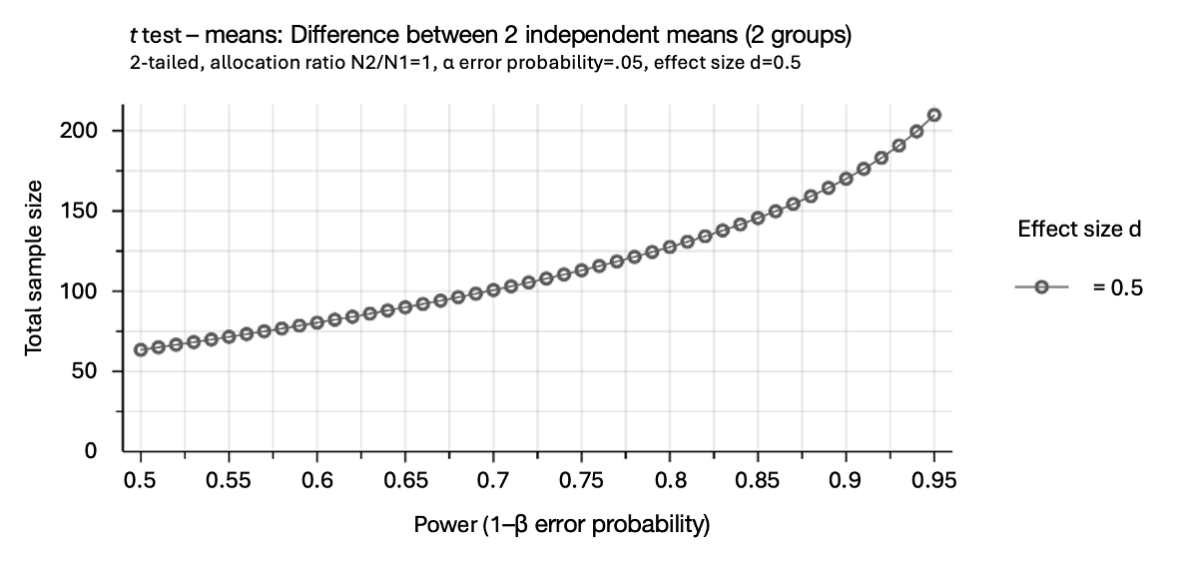
Power analysis for between-group comparison (GPower).

A post hoc power analysis was conducted to assess the statistical power of the study based on the observed sample size of 100 participants (50 per group). Assuming a moderate effect size (Cohen *d*=0.50) and an α level of .05, the analysis indicated that the study has 0.7 power to detect between-group differences as visualized in Figure 2.

### Inclusion and Exclusion Criteria

**Figure 3:**
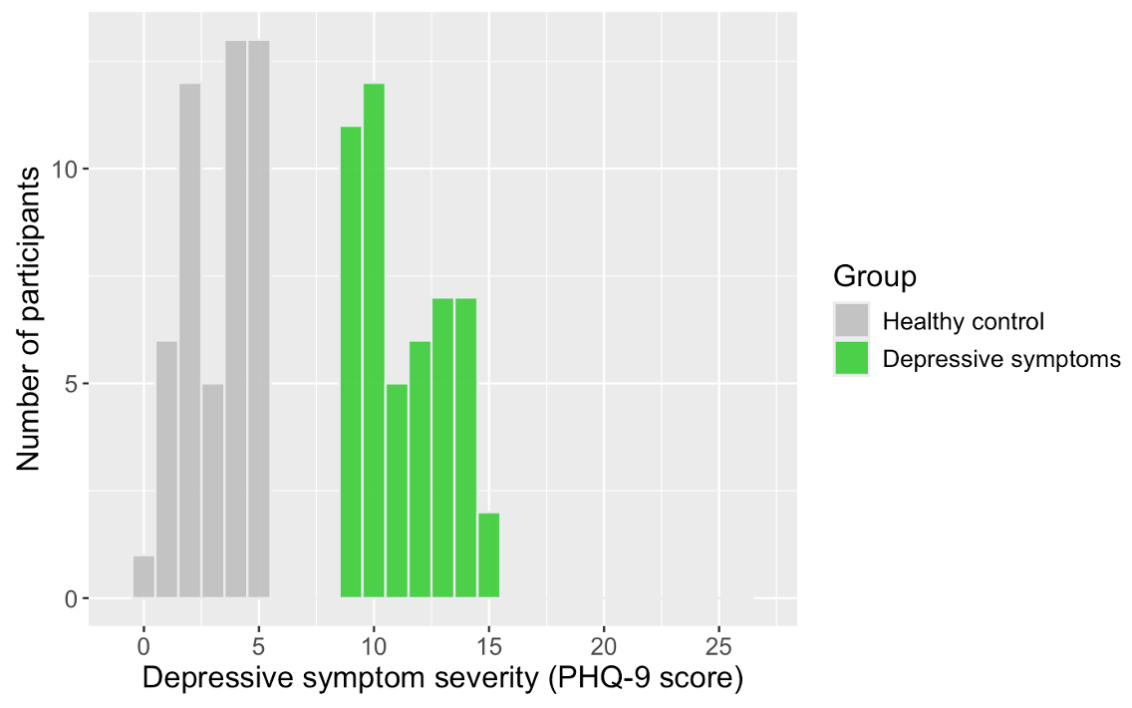
Distribution of participants’ scores on PHQ-9 assessing current depressive symptom severity. PHQ-9: Patient Health Questionnaire-9.

The screening for inclusion involved the assessment of current DS severity using the Patient Health Questionnaire-9 (PHQ-9; Kroenke et al [26]). Participants scoring 9 or above—constituting the upper limit for mild DSs [26]—were assigned to the DS, while those scoring 5 or below were registered for the HC group (Figure 3).

For inclusion in either group, participants had to be free from any condition that would impair their ability to interact with the VR environment (eg, visual impairment without the possibility of correction via lenses) and able to provide written informed consent. Additional inclusion criteria for the DS group included the lack of diagnosis of any other psychiatric disorders than depressive or anxiety disorders, and treatment stability over the last 4 weeks if they received psychological or pharmacological treatment at the time of assessment. Participants in the control group had to be free from any previous or current psychiatric disorders.

### Study Procedure

After providing informed consent and completing the pre-VR battery questionnaires, all participants were seated in a room equipped with the VR system. Following a headset and eye-tracking calibration, a tutorial was completed (aimed to help the understanding of how to move around and interact with the environment). Next, participants were free to explore the VR environment on their own terms but in the presence of a study administrator. The study session was concluded once the post-VR battery was completed.

### Technological Specifications

#### Overview

The VR system was developed using the HP Reverb G2 Omnicept Edition headset, which includes a variety of biometric sensors such as eye tracking, a facial camera (lower), a heart rate sensor, and sensors for brain activity while maintaining the core features of the standard version. These features include a resolution of 2160 × 2160 px per eye, a 114° field of view, a 90 Hz refresh rate, and integrated audio.

#### Environment Development

The virtual environment was developed entirely in Unity 2020.3.39 LTS, using the HP Omnicept SDK and OpenXR to maximize compatibility and platform versatility. Several development processes were used to ensure a smooth and efficient environment, including occlusion culling, foveated rendering, baked lighting, and asset optimization.

The scenario consists of 4 rooms, each with distinct lighting parameters, as well as an additional room that can be visually explored through a keyhole in a door. Volume postprocessing was used to modify the lighting without affecting performance, and the keyhole effect was simulated using camera layers.

Within each room, various stimuli and interactive elements are present, such as sound boxes, a chalkboard, and interactable objects (eg, items that can be picked up and thrown, musical buttons, and photos that can be picked up for closer inspection). Every interaction between the participant and the environment is recorded, including eye tracking data, particularly in relation to elements designated as areas of interest or with which the subject can interact (eg, ENTERING_ROOM, LEAVING_ROOM, TAKE, DROP, TRIGGER, INTERACT).

#### Administrator Control

To manage the experiment, the study administrator is provided with an interface, enabling them to observe the participant’s actions in real-time. The interface also offers various options to ensure that the experimental session proceeds smoothly (eg, it is possible to discontinue a cognitive task if the participant requests so). This control system is facilitated by an additional camera mounted on the subject, enabling the administrator to monitor the experiment’s progression effectively.

### Virtual Environment

The goal of the pilot VR environment is to differentiate between HCs and those with DSs based on cognitive, behavioral, and physiological data. The development and performance of the system for this purpose will be discussed in more detail in another paper [25], but the main characteristics will be summarized here for a general overview.

The environment was designed to elicit certain behaviors and record measures, some of which were previously shown, and others are hypothesized to be connected to depression [25]. The domains included cognition (attention, working memory, processing speed, executive functioning, and cognitive flexibility), metacognition, persistence or grit, curiosity, as well as behavioral and attentional biases toward negative stimuli, speed, and movement measurements. Physiological data included skin conductance, heart rate variability, and eye-tracking–related measures.

**Figure 4:**
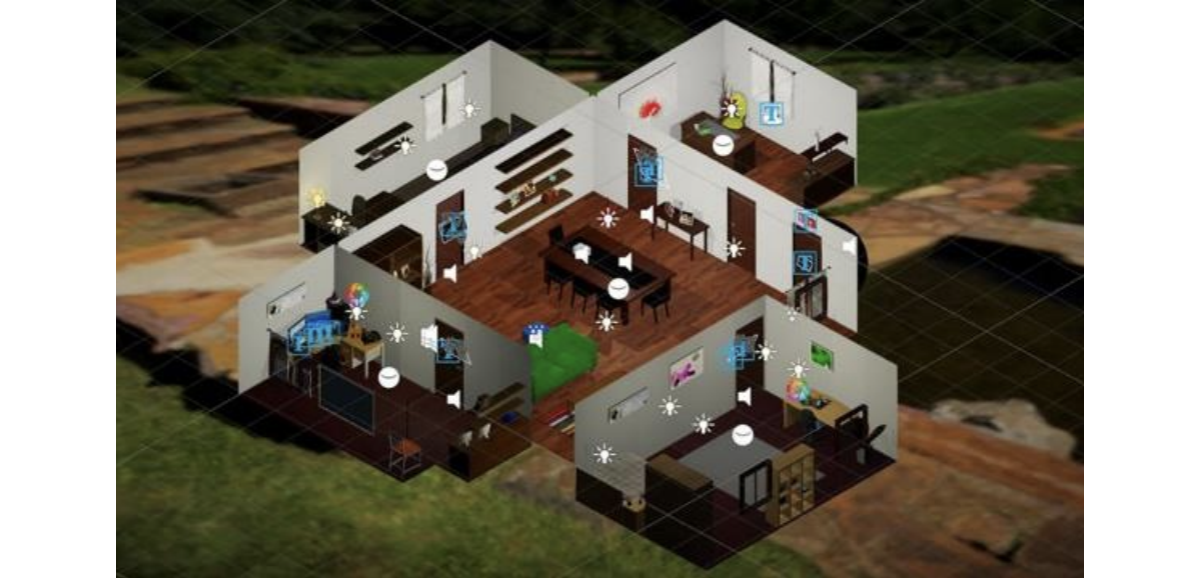
The visual illustration of the virtual reality environment designed for the assessment of cognition and behavior hypothesized to be related to depression.

The environment resembled a multiroom family home (Figure 4). Exposure started with a tutorial introducing participants to controllers as well as how to move around in the environment and interact with objects. Following the completion of the tutorial, participants were free to explore the environment but had to solve cognitive tasks to open doors and move between rooms. There were 4 cognitive tasks in total: Rapid Visual Information Processing; N-back [2-back]; Trail Making Test—parts A and B; and Wisconsin Card Sorting Test. The exposure ended on the participant’s own terms, but the exit door only opened once all cognitive tasks were completed.

Multimodal data collected during the VR sessions for the purpose of DS assessment were analyzed using a CatBoost machine learning algorithm (Yandex LLC) [27]. Model performance was evaluated based on accuracy, and feature importance was determined through an explainable artificial intelligence approach [28], ensuring transparency in the decision-making process. For more details, see the study by Mura et al [25].

### Tools and Questionnaires

#### Pre-VR Questionnaires

The pre-VR battery included a background questionnaire (demographic data and diagnosis, medication); the Patient Health Questionnaire (PHQ-9) [26] to assess current DS severity; the Depression, Anxiety, Stress Scale (DASS) [29] to assess current depressive, anxiety and stress symptom severity; the Positive and Negative Affect Schedule (PANAS-SF) [30] to evaluate emotional state; as well as the Curiosity and Explorations Inventory-II [31].

#### Post-VR Questionnaires

The post-VR battery repeated the PANAS-SF questionnaires and included questionnaires regarding, acceptability, usability, and cybersickness, described in more detail in the later sections.

#### Acceptability

The construct of acceptability was assessed based on the theoretical framework of acceptability (TFA) [18,32]. The acceptability of the concept and the acceptability of the pilot system were evaluated separately. The acceptability of the concept, that is, the use of VR for the purposes of mental health screening, diagnosis, and treatment (definitions were provided) was assessed via self-report Likert items. Scores ranged from 1 to 5, indicating answers from “Strongly disagree” to “Strongly agree” on statements about VR being acceptable (Multimedia Appendix 1). As the primary purpose of the pilot system was the assessment of DSs, the diagnostic use case was investigated via 2 additional questions.

- Support tool: Willingness to engage with the system to inform diagnosis made by a mental health professional.
- Stand-alone tool: Willingness to engage with the system to receive a diagnosis without extra input from a mental health professional.

The dimensions of acceptability concerning the pilot system were assessed within the TFA and focused on general acceptability, affective attitude, perceived effectiveness, intervention coherence, self-efficacy, burden, ethicality, and opportunity cost. The construct of affective attitude was further broken down into two questions: (1) To what extent a participant likes or dislikes the system, and (2) how comfortable or uncomfortable they find the system to be. For ease of interpretability, the rating scale was unionized across all constructs, where “1” represented the “undesirable” rating (eg, low levels of comfort; or the need for high levels of effort indicated burden), and “5” reflected the “desirable” rating (eg, high levels of comfort; or low levels of effort reported under burden). See Multimedia Appendix 1 for details about the specific items used to assess each of these constructs.

#### Usability

Usability was assessed by the System Usability Scale ​(SUS) [33]—which is a postinteraction self-report questionnaire to assess user perceptions of a system’s overall usability. The scale is composed of 10 items rated on a Likert scale from 1 to 5, from “strongly disagree” to “strongly agree.” The total score ranges from 0 to 100, with higher scores indicating higher usability ratings. There are multiple frameworks to interpret the SUS score (range, adjective, and grade) [34], but the primary threshold for this study was the average score necessary for an ‘OK/satisfactory’ experience (a score of 50.9 [35]).

#### Cybersickness

To assess levels of cybersickness following VR exposure, the Simulator Sickness Questionnaire (SSQ) was used [36]. The questionnaire contains 16 symptoms, which are rated on a scale of subjective severity: 0=none, 1=slight, 2=moderate, and 3=severe. Beyond the total score, one can attain separate scores for the nausea, oculomotor disturbances, and disorientation subscales.

However, while the SSQ was once considered to be the “gold standard,” it has increasingly been criticized, including the original thresholds for the interpretation of the severity levels [37-40]. For this reason, the comparison of the pilot system results was made to the literature average [23] instead of the original thresholds.

### Analysis

All analyses were carried out in R supplemented by R Studio (version 2023.12.1.402; R Foundation for Statistical Computing), except for the power calculation for which G*Power (version 3.1; Heinrich Heine University Düsseldorf) was used. Multiple packages were used to supplement analysis and visualization, these include but are not limited to: *dplyr* [41], *vioplot* [42], *ggplot2* [43], *psych* [44], *rstatix* [45], *ggeffects* [46], *ordinal* [47], and *VGAM* [48]. See Multimedia Appendix 2 for the script, its output, and a full list of packages.

Within-group comparisons and comparisons to known literature values were conducted using Wilcoxon signed rank tests, while between-group comparisons used Mann-Whitney *U* tests. These nonparametric methods were chosen to account for the ordinal nature of the outcome measures, such as Likert scales, and to address the observed violations of the assumptions required for parametric tests (eg, normality assessed via the Shapiro-Wilk test). A nominal significance level (α) of .05 was applied. For ease of interpretability, effect sizes were consistently reported in *r* values. This includes the use of rank-biserial correlations for Mann-Whitney *U* tests as well as Wilcoxon signed rank tests for quantifying the magnitude of the differences in a standardized manner. The full statistics are available in Multimedia Appendix 2.

Multivariate linear regression models were used to analyze predictors when the outcome variable was continuous, such as SUS or SSQ scores. For ordinal outcome measures, such as TFA constructs, proportional odds models were applied. In cases where the proportional odds assumption was not met (as noted in the Results section), results should be interpreted with caution. All regression models included age, sex, and education as covariates. When additional predictors, such as cybersickness severity scores, group labels, or self-reported anxiety and stress levels were relevant, these were included alongside the demographic variables.

### Ethical Considerations

The study was carried out in accordance with the Declaration of Helsinki and obtained ethical approval from the local ethical committees on all sites where data was collected (Italy) or analyzed (Italy and Sweden). In Italy, approval was granted by Comitato Etico della Ricerca Psicologica, and in Sweden by the Etikprövningsmyndigheten (2023-00959-01).

Written informed consent was sought from all participants, with emphasis placed on the voluntary nature of participation and the possibility to withdraw from the study at any time, without reason, or any penalty. All participants were informed about data privacy and confidentiality, whereby all data obtained during the study were analyzed anonymously and used solely for the purpose of scientific research. Compensation included €25 (worth between US $24.38 and US $28.14 over the duration of the recruitment period).

### Preregistration

The study was preregistered in the ISRCTN registry (ISRCTN16396369) [49]. The sole modification to the preregistered protocol concerned the threshold for inclusion on current DS severity. Due to the difficulty in recruiting volunteers in line with the study’s timeline, the upper threshold for inclusion in the control group was increased from 4 to 5, while the lower threshold to be included in the group with DSs was decreased from 10 to 9 on the PHQ-9.

## Results

### Acceptability of the Concept

**Figure 5:**
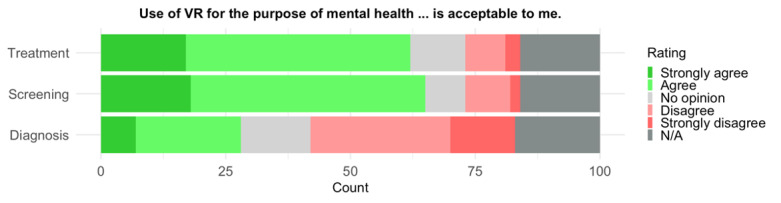
Participant ratings of VR acceptance for the purposes of mental health screening, diagnosis, and treatment. N/A: not available; VR: virtual reality.

The use of VR for mental health treatment (62/84, 74%) and screening (65/84, 77%) was endorsed by the majority of those providing an answer, whereas only a minority indicated support for diagnostic purposes (28/83, 34%; Figure 5). There were no significant differences between the DS and HC groups.

**Figure 6:**
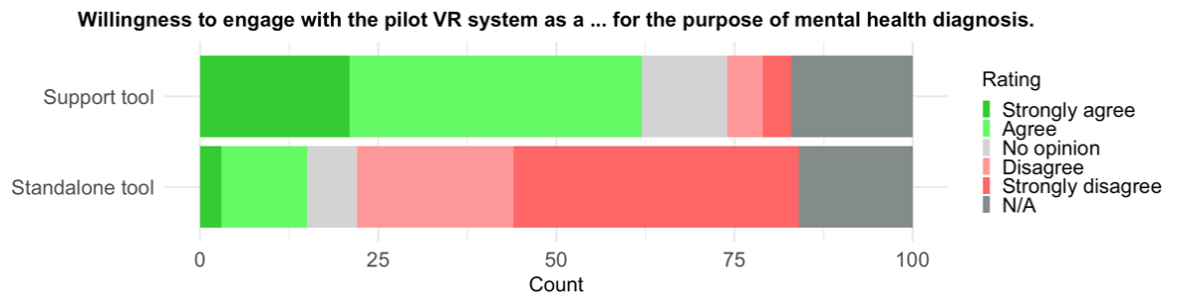
Participants’ subjective ratings of their willingness to engage with the pilot VR system for mental health diagnosis. The support tool complements the assessment of a mental health professional, whereas the standalone tool operates without professional input. N/A: not available; VR: virtual reality.

Comparing the use of VR with or without input from a mental health professional, the responses indicate higher support for the VR system to be used as a support tool (62/83, 75%) compared to a stand-alone solution (15/84, 18%). A Wilcoxon signed rank test indicated a significant difference of large size (*V*=1770; *P*<.001, 2-tailed; *r*=0.81, 95% CI 0.76-0.84; median_support_ 4, IQR_support_ 3.5-4.5; median_stand-alone_ 2, IQR_stand-alone_ 1.0-3.0). For the raw distribution of responses see Figure 6. Again, no difference was observed between the DS and HC groups.

### Acceptability of the Pilot System

Behavioral measures of acceptability include the discontinuation of participation for any reason, as well as the need to take a break from the study. While the possibility to discontinue participation at any point, without any consequences was stated before enrollment, all 100 volunteers completed the study. The need for a break was expressed by 1 participant (1/100, 1%) due to discomfort. They decided to resume participation despite the possibility of opting out being reemphasized. On average, 31 minutes were spent in the virtual environment.

**Figure 7:**
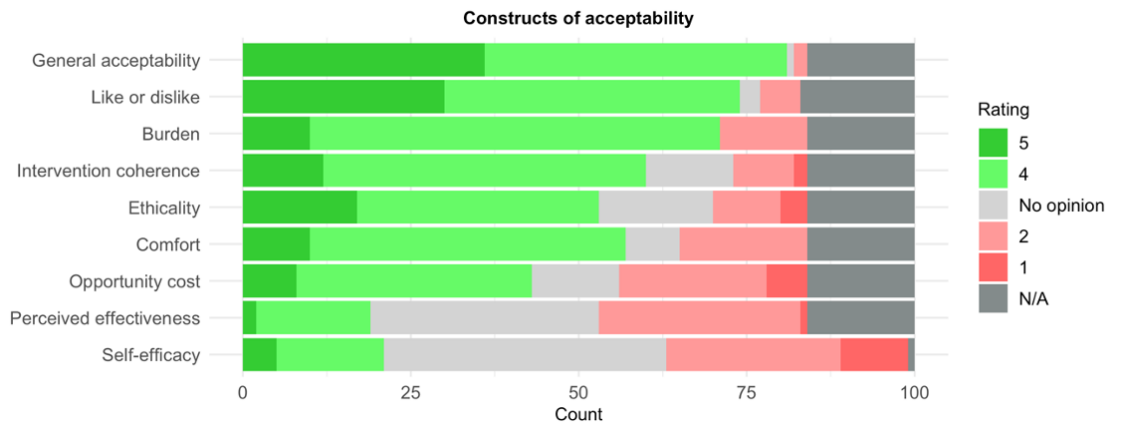
Self-reported acceptability of the pilot virtual reality system, assessed using constructs from the theoretical framework of acceptability (1=undesirable rating; 5=desirable rating). N/A: not available.

Acceptability was also assessed via a self-report questionnaire specifically formulated for the project based on the TFA [18,32]. Ratings, visualized in Figure 7, indicated generally high levels of acceptability.

A majority of the participants found the system itself acceptable (81/84, 96%), liked the system (74/83, 89%), indicated that the engagement took little to no effort (71/84, 85%), found it clear how the system might help diagnose depressive disorders (60/84, 71%), found no moral or ethical consequences with the system (53/84, 63%), found it comfortable (57/84, 68%), and expressed no concern about missing out on other alternatives when engaging with the system (43/84, 51%). The 2 constructs on which the undesirable ratings (1 or 2) outnumbered desirable scores (4 or 5) concerned the perceived effectiveness of the system in improving mental state (19/84, 23%), and self-efficacy regarding feeling confident while engaging with the system (21/99, 21%).

Multivariate ordinal regression models were used to explain variance in ratings of all the acceptability constructs as detailed in Table 2. Sex was a significant predictor of general acceptability and subjective burden, where women reported considerably lower levels of acceptability (mean_men_ 4.65, SD_men_ 0.49; mean_women_ 4.30, SD_women_ 0.65) and reported lower ratings on burden—meaning a considerably higher effort was required from their part to engage with the system (mean_men_ 4.29, SD_men_ 0.50; mean_women_ 3.69, SD_women_ 0.87). Education did not reach significance for any of the TFA constructs, and age was marginally significant only when used to explain whether participants found moral or ethical consequences to engaging with the system.

In the next stage, group label (DS vs HC) was entered into the same models in addition to the demographic variables. Group label was a significant predictor of comfort levels (mean_HC_ 3.83, SD_HC_ 0.78; mean_DS_ 3.34, SD_DS_ 1.08) and marginally significant for the like or dislike rating (mean_HC_ 4.38, SD_HC_ 0.59; mean_DS_ 4.00, SD_DS_ 0.95)—meaning that participants in the DS group reported lower comfort levels and liked the system to a lesser extent.

Finally, cybersickness severity (total SSQ score) was entered into models explaining all TFA constructs, in addition to the demographic variables. Cybersickness was a significant predictor for comfort, burden, opportunity cost, and perceived effectiveness and marginally significant for self-efficacy (Table 2). All showed an inverse relationship, whereas the higher the cybersickness symptom severity, the lower acceptability scores were reported on all TFA dimensions.

In summary, both the concept and the system received high acceptability ratings. Most participants supported the use of VR for mental health screening and treatment. However, regarding diagnosis, participants preferred VR as a supportive tool rather than a stand-alone solution. The primary area for improvement identified was enhancing users’ self-efficacy while interacting with the system. Sex and cybersickness emerged as significant predictors of acceptability ratings.

**Table 2:** Estimates of predictors from 3 different models analyzed via multivariate ordinal regressions to explain constructs investigated within the theoretical framework of acceptability.

Model				Predictor						Estimate (log odds)						95% CI						*P* value				
**General acceptability**																										
	Model 1							Age						0.175						–0.19 to 0.56						.36
	Model 1							Sex						–1.331						–2.54 to –0.21						.02^a^
	Model 1							Education						–0.529						–1.10 to 0.01						.053
	Model 2							Group (DS^b^ vs HC^c^)^d^						–0.261						–1.19 to 0.65						.58
	Model 3							Cybersickness						–0.004						–0.02 to 0.01						.54
**Like or dislike**																										
	Model 1						Age						–0.298						–0.67 to 0.05						.10	
	Model 1						Sex						–0.461						–1.52 to 0.58						.39	
	Model 1						Education						0.164						–0.34 to 0.67						.52	
	Model 2						Group (DS vs HC)^d^						–0.890						–1.81 to 0.00						.053	
	Model 3						Cybersickness						–0.010						–0.03 to 0.01						.20	
**Comfort**																										
	Model 1						Age						–0.045						–0.38 to 0.29						.79	
	Model 1						Sex						–0.718						–1.84 to 0.37						.20	
	Model 1						Education						0.116						–0.36 to 0.58						.63	
	Model 2						Group (DS vs HC)^d^						–0.899						–1.81 to –0.02						.048^a^	
	Model 3						Cybersickness						–0.026						–0.04 to –0.01						.001^e^	
**Burden**																										
		Model 1	Age						0.137						–0.26 to 0.54						.50					
		Model 1	Sex						–1.866						–3.26 to –0.59						.005^e^					
		Model 1	Education						–0.186						–0.78 to 0.38						.53					
		Model 2	Group (DS vs HC)^d^						–0.273						–1.28 to 0.72						.59					
		Model 3	Cybersickness						–0.036						–0.06 to –0.02						<.001^f^					
**Intervention coherence**																										
	Model 1					Age						0.085						–0.25 to 0.42						.62		
	Model 1					Sex						–0.315						–1.42 to 0.77						.57		
	Model 1					Education						–0.193						–0.69 to 0.29						.44		
	Model 2					Group (DS vs HC)^d^						–0.567						–1.44 to 0.29						.20		
	Model 3					Cybersickness						–0.003						–0.02 to 0.01						.71		
**Ethicality**																										
	Model 1					Age						0.320						–0.00 to 0.65						.053		
	Model 1					Sex						0.241						–0.74 to 1.23						.63		
	Model 1					Education						–0.166						–0.66 to 0.33						.51		
	Model 2					Group (DS vs HC)^d^						0.170						–0.63 to 0.98						.68		
	Model 3					Cybersickness						–0.010						–0.02 to 0.00						.15		
**Opportunity cost^g^**																										
	Model 1		Age						–0.078						–0.39 to 0.24						.63					
	Model 1		Sex						–0.496						–1.53 to 0.50						.34					
	Model 1		Education						0.023						–0.44 to 0.49						.92					
	Model 2		Group (DS vs HC)^d^						0.336						–0.46 to 1.14						.41					
	Model 3		Cybersickness						-0.019						–0.03 to –0.01						.009^e^					
**Perceived effectiveness**																										
	Model 1				Age						–0.116						–0.44 to 0.20						.48			
	Model 1				Sex						–0.757						–1.79 to 0.25						.14			
	Model 1				Education						–0.045						–0.53 to 0.43						.85			
	Model 2		Group (DS vs HC)^d^						–0.548						–1.38 to 0.27						.19					
	Model 3		Cybersickness						–0.019						–0.04 to –0.00						.02^a^					
**Self-efficacy^h^**																										
	Model 1				Age						0.063						–0.24 to 0.37						.68			
	Model 1				Sex						–0.872						–1.86 to 0.08						.08			
	Model 1				Education						0.031						–0.40 to 0.48						.89			
	Model 2		Group (DS vs HC)^d^						0.043						–0.70 to 0.79						.91					
	Model 3		Cybersickness						-0.01						–0.03 to 0.00						.08					

^a^*P*<.05.

^b^DS: depressive symptom.

^c^HC: healthy control.

^d^The categorization is based on current depressive symptom severity assessment via the PHQ-9 questionnaire (score equal to 9 or above for depressive symptoms [DS] and 5 or below for healthy controls [HC]) and not the presence of a clinical diagnosis.

^e^*P*<.01.

^f^*P*<.001.

^g^The assumption of proportional odds not was not fulfilled for the model. Results should be interpreted with caution.

^h^Self-efficacy was measured with Item 9 of the System Usability Scale [33], as opposed to the other items that were specifically designed for the assessment of the pilot system based on the theoretical framework of acceptability [18,32].

### Usability

#### Descriptives

**Figure 8:**
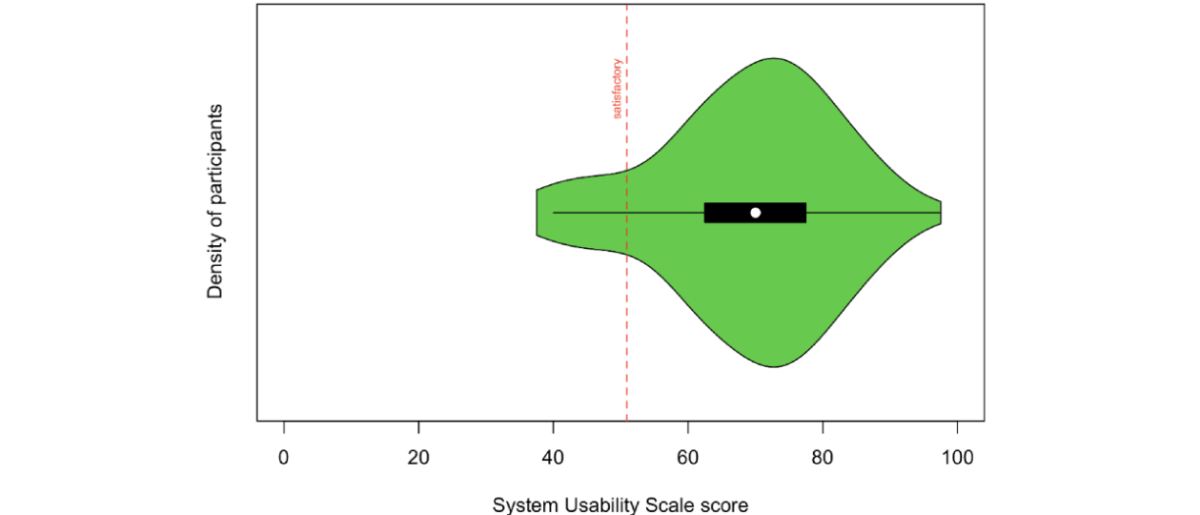
Density plot of overall usability ratings on the System Usability Scale from all participants.

Results from 99 participants indicated average ratings of usability, with a mean score of 69 (SD 12.86), and a range of 37.5-97.5 on SUS [33]. As visualized in Figure 8, which showcases the distribution of overall SUS scores, 88% (87/99) of participants provided a rating of 50.9 or above, indicating an OK or satisfactory experience or better [35].

#### Analysis

A multiple linear regression analysis was conducted to predict usability ratings based on participants' age, education, gender, and group (DS vs HC). The regression model was not statistically significant (*F*_4,94_=1.23; *P*=.31), explaining only about 5% of the variance in SUS scores (R^2^=0.05, adjusted R^2^=0.01). The strongest predictor was gender, but this only showed a marginal effect (β=–5.79, 95% CI –12.38 to 0.80; *P*=.08) with men reporting higher usability ratings than women.

A second model was tested examining the relationship between usability scores and the predictors of age, education, sex, and total cybersickness severity. The model was significant (*F*_4,94_=2.97; *P*=.02), accounting for 11.2% of the variance in SUS scores (R^2^=0.11, adjusted R^2^=0.07). Cybersickness severity (SSQ scores) emerged as the only significant predictor (β=–0.12, 95% CI –0.21 to –0.03; *P*=.007), indicating that participants with higher SSQ scores tended to have lower SUS scores.

In summary, the system’s overall usability was rated as sufficient, with the majority of participants reporting a satisfactory experience. Cybersickness severity emerged as the only predictor of usability ratings, showing a negative association.

### Cybersickness

#### Descriptives

The overall mean SSQ score combining all subscales was 30.49 (SD 28.03), with individual scores ranging from 0 to 119.68. This is comparable to the literature average of 28.00 reported by Saredakis et al [23], on the overall sample, as well as the DS subsample. However, the HC group reported considerably lower levels of cybersickness severity (V=402; *P*=.02; *r_rb_*=–0.37, 95% CI –0.61 to –0.07) as summarized in Table 3.

For the SSQ subscales, the results of the total sample reflect severity levels not (significantly) different from the literature average [23] regarding nausea and disorientation levels, whereas oculomotor disturbances were rated considerably higher in this study (V=3296; *P*=.004; *r_rb_*=0.33, 95% CI 0.12-0.52).

**Figure 9:**
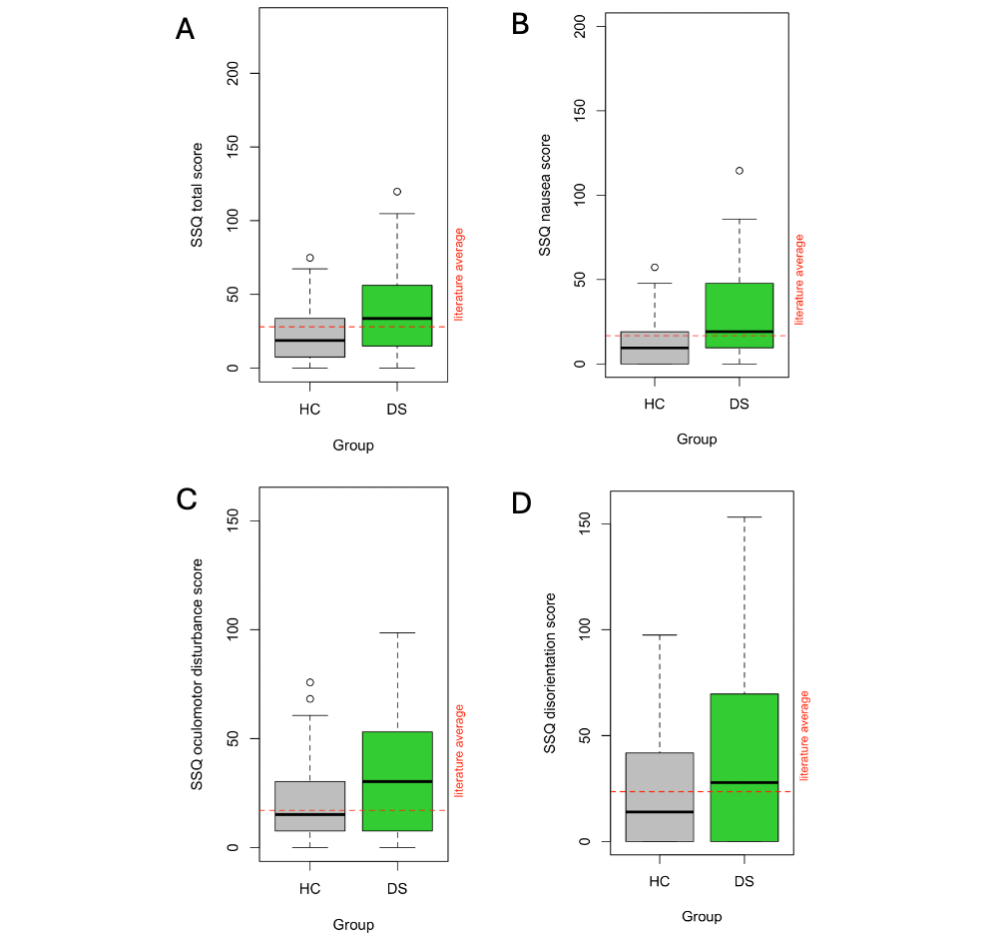
Boxplot of self-reported cybersickness severity scores for the HC and DS study groups—measured by the (A) SSQ, and its 3 subscales of (B) nausea, (C) oculomotor disturbances, and (D) disorientation. DS: depressive symptom; HC: healthy control; SSQ: Simulator Sickness Questionnaire.

When the comparison was split by study groups, only the DS group deviated significantly from the literature average—specifically on the nausea (V=917; *P*=.002; *r_rb_*=0.50, 95% CI 0.22-0.70) and the oculomotor disturbances (V=967; *P*<.001; *r_rb_*=0.58, 95% CI 0.33-0.75) subscales. The HC group showed near-average levels on all 3 subscales. The distributions of raw scores, as boxplots, along the total and all subscales are illustrated in Figure 9 and analyzed for between-group differences in the later section.

**Table 3:** Comparison of mean cybersickness severity scores (SSQa; mean [SD]) via Wilcoxon-signed rank tests across the total sample, those with DSb and HCc subsamples with literature averages [23].

SSQ Scale	Literature average^d^	Total sample, mean (SD)	DS^e^, mean (SD)	HC, mean (SD)
Total	28.00	30.49 (28.03)	39.39 (32.89)	21.77 (18.84)^f^
Nausea	16.72	21.59 (21.96)	29.40 (24.39)^g^	13.93 (16.15)
Oculomotor disturbances	17.09	26.72 (24.63)^g^	33.57 (27.50)^h^	20.01 (19.48)
Disorientation	23.50	32.90 (39.84)	42.04 (48.34)	23.94 (26.83)

^a^SSQ: Simulator Sickness Questionnaire.

^b^DS: depressive symptom.

^c^HC: healthy control.

^d^Literature averages are taken from Saredakis et al [23] based on a meta-analysis of 55 studies on approximately 3000 participants.

^e^The categorization is solely dependent on the Patient Health Questionnaire-9 score and does not indicate the presence of a clinical diagnosis.

^f^*P*<.05.

^g^*P*<.01.

^h^*P*<.001.

#### Analysis

A multiple linear regression was conducted to predict SSQ scores based on age, education, sex, and group (DS vs HC). The model was statistically significant (*F*_4,94_=3.20; *P=*.02), accounting for approximately 12% of the variance in SSQ scores (*R^2^*=0.12, adjusted *R^2^*=0.08). The only predictor reaching significance was group label, (β=17.58, 95% CI 6.57-28.60; *P*=.002), indicating that participants in the DS group had considerably higher SSQ scores (mean 39.38, SD 32.89) than the HC group (mean 21.77, SD 18.84). These between-group differences remained significant for all subscales analyzed separately.

**Figure 10:**
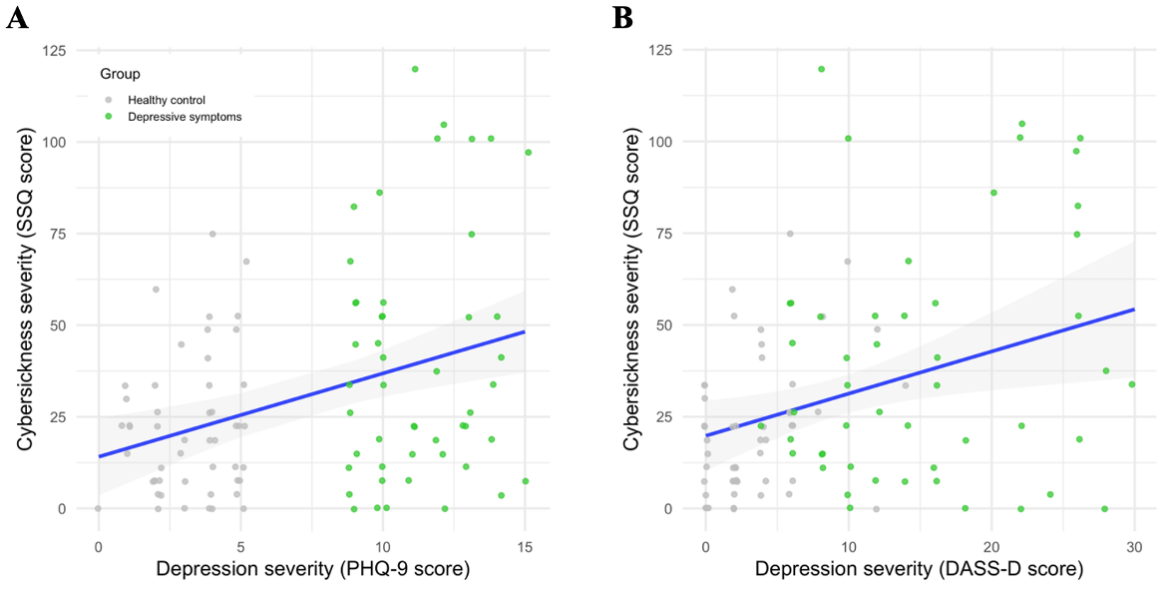
Predicted cybersickness severity levels (with 95% CIs) based on depressive symptom severity, as measured by: (A) PHQ-9 and (B) DASS. DASS: Depression Anxiety Stress Scale; DASS-D: Depression Anxiety Stress Scale-Depression; PHQ-9: Patient Health Questionnaire; SSQ: the Simulator Sickness Questionnaire.

When DS severity was entered as a continuous variable instead of a group label, a further 2% variance in cybersickness severity was explained (*R^2^*=0.14, adjusted *R^2^*=0.11) and a linear prediction of SSQ scores along the PHQ-9 scores was attainable as visualized in Figure 10A. The graph shows all participants along the SSQ and PHQ-9 scales and illustrates a positive association between the 2 scores when the regression line is plotted.

However, due to the baseline group differences in anxiety and stress levels (Table 1) this prediction might be biased. While DASS depression was not of primary interest, a multiple linear regression model with DASS depression as an outcome was used, to be able to adjust for DASS anxiety and DASS stress levels in addition to age, sex, and education levels as covariates. This model was also significant (*F*_6,92_=4.05; *P=*.001) and explained about 21% of the variance in cybersickness severity (*R^2^*=0.21, adjusted *R^2^*=0.16). The only predictor emerging as significant was DASS depression (β=1.15, 95% CI 0.29-2.01; *P=*.009), whereas DASS anxiety reached marginal significance (β=1.2, 95% CI –0.07 to 2.47; *P*=.06). Cybersickness severity predictions showed a similar trend as for the PHQ-9 scores when the regression line was illustrated in Figure 10B.

In summary, the severity of cybersickness was comparable to the literature averages for nausea and disorientation but significantly higher for oculomotor disturbances, particularly in the DS group. Across all subscales, the DS group reported higher SSQ scores than the HC group, with group membership emerging as the strongest predictor of cybersickness severity. A positive association was identified between DS severity (PHQ-9) and cybersickness, explaining a modest but significant proportion of variance. Even after adjusting for baseline anxiety and stress levels (DASS), DSs remained a significant predictor of cybersickness.

## Discussion

### Principal Findings

This study reports on the acceptability, usability, and cybersickness levels of a novel VR environment designed for the assessment of DSs. Following an approximately 30-minute engagement, 50 HCs and 50 individuals with moderate self-reported DSs completed a self-report battery on 3 constructs of interest.

### Acceptability

In general, VR systems were rated acceptable by the majority of participants; both for the purpose of mental health screening as well as treatment. For diagnostics, a clear preference emerged for VR to be used as a support tool by a health care professional, as opposed to a stand-alone solution. While there is no literature available on VR for depression with which a direct comparison can be made, these findings are in line with previous results concerning other digital solutions, such as mHealth apps [50].

The specific pilot system tested here was designed for the purpose of differentiating between HCs and those with DSs [25]. The system itself was rated acceptable by most participants, but the need for improvement was apparent; specifically when it came to participants’ confidence levels while using the system. Nevertheless, the high level of acceptance in practice found in the current study is in line with 2 studies previously reported on the acceptability of specific VR systems for therapeutic purposes for depression [21,22].

### Usability

Usability was rated satisfactory by most of the participants, with cybersickness severity emerging as a significant predictor of usability scores. We found 1 previous study reporting on the usability of VR systems for depression, which has also indicated favorable ratings on ease of use and perceived usefulness [21]. However, in comparison with other psychiatric conditions, such as social anxiety disorder [51], psychosis [52], or posttraumatic stress disorder [53], it is important to emphasize that systematic evaluations of the usability of VR among those with depression are lacking.

Concerning the effect of cybersickness severity on usability, there are no previous studies reporting in relation to depression. Additionally, while comprehensive reviews on the relationship between cybersickness and presence are available [54] there is no such overview for usability. Existing single studies report inconsistent findings across different conditions. For example, a negative association is reported on healthy individuals [55], but no effect was found among those with Huntington disease [56].

These findings highlight the need for greater attention from the scientific community, as depression remains relatively understudied in the context of VR use compared to other psychiatric conditions [12,57]. Notably, participants in this study expressed support for the technology both conceptually and in relation to this specific pilot system. Identifying novel approaches with user support is particularly important for groups that might find treatment adherence and lack of motivation to engage challenging, such as those with depression [58].

The high levels of acceptance and usability that users have shown toward VR technology within the study, both in theory and practice, lay a solid ground for investigations into how VR could be used in a mental health context. VR being a premise for real-time data collection via direct observation of behavior contributes to higher ecological validity and allows for the consideration of a wider set of variables when assessing mental states [6,7]. While the potential is promising, reviews of real-life studies [59], editorials [60] as well as commentaries [57], highlight a need for further scientific investigation, to better inform how VR is best implemented within clinical practice.

### Cybersickness

Finally, the results of the study show significant differences in self-reported cybersickness levels between HCs and those with DSs —the latter reporting considerably higher levels. This difference brings forward many questions, some relating to the self-report instrument used, as well as its administration; these will be discussed under the limitations more thoroughly. Beyond the critical appraisal of the instrument and optimizing the system so that we minimize the risk of cybersickness in general [61-63], this difference urges a consideration of how cybersickness might relate to depression. It is important to consider that our study lacked a pre-post comparison, which creates challenges when attempting to disentangle cybersickness symptoms evoked by VR exposure from potential baseline differences stemming from somatic symptoms related to depression. While “the language used in the medical literature to describe somatic symptoms in depression is both confusing and contradictory” [64], one can pinpoint constructs related to depression, that may also be captured by cybersickness scales—which may cause contamination when assessing cybersickness levels. These include, but might not be limited to headache, fatigue, nausea, dizziness, and gastrointestinal disturbances [36,64]. This hypothesis needs further testing but is in line with the only study reporting on SSQ in a pre-post manner among those with psychiatric conditions including depression (heterogeneous sample of anxiety, psychotic, depressive, or bipolar disorder)—where the baseline levels of cybersickness would already be classed as “severe,” but there is no significant elevation reported post exposure [65].

Nonetheless, it is important to consider that the elevated cybersickness in the group with DSs could still be, at least in part, a response to the VR environment itself. If this is the case, using VR with the right study design may serve as a potential tool for further exploring the sensory abnormalities in depression, though ethical considerations are warranted. Investigating the underlying mechanisms of cybersickness in depression may also provide novel insights into the somatic manifestations of the condition and could help further define depression as well as refine interventions for mental health. Additionally, the need to consider cybersickness severity becomes even more apparent in light of the results showing its association with acceptability and usability ratings, which might transfer into the real-life uptake and use of VR technology.

### Limitations and Future Directions

The study is not without its limitations. First, one has to consider the characteristics of the participants, as the sample consisted of young adults, the majority being women, and highly educated which limits generalizability [66]. In general, lower age and higher education levels are associated with increased digital health literacy [67]. However, further reports are needed to understand how such sample characteristics—sociodemographic variables as well as DSs—influence user attitudes to digital technologies and engagement in a mental health context [68,69]. To this aim, there is a clear need for extending the research to include diverse age groups and educational backgrounds to enhance the generalizability and applicability of the findings.

Second, the categorization between the HC and the DS groups was based solely on self-reported symptoms at the time of the study (using the PHQ-9) as opposed to the presence or absence of a clinical diagnosis. Other forms of assessment might be worthy of consideration. Additionally, the majority of individuals labeled as DS only reported moderate depression severity, which questions whether the results are generalizable to those with more severe symptoms. Self-report measures in general are subject to response biases, such as social desirability, or recall bias. The subjective evaluation of symptom severity in this case could potentially have resulted in over- or underreporting of severity.

Third, although we controlled for demographic covariates, baseline differences in anxiety and stress levels between the study groups may have impacted our results. Self-reported stress and anxiety significantly differed between the HC and DS groups, highlighting that between-group differences in our constructs of interest cannot unequivocally be attributed to differences in DS severity.

Fourth, the measurement of cybersickness levels leaves room for improvement on multiple fronts. The instrument (SSQ) has repeatedly been criticized for reasons among others being the low power to differentiate from anxiety and the questionable assumption of having zero “symptoms” at baseline [37-40]. The difference in severity between the HCs and those with DSs cannot unequivocally be attributed to the VR exposure, as no baseline levels were recorded. It is possible, that at least some degree of the difference originates from baseline differences in anxiety or somatic symptoms of depression, which the questionnaire most likely does not adequately differentiate from cybersickness symptoms. In the future, assessment of related symptoms necessitates pre-post comparisons, particularly relevant when the SSQ is being used [37-40].

Fifth, when creating items to assess constructs of the TFA, 1 item was taken from the SUS questionnaire (item 9: confidence), while all other items were specialized to this study. Overall, single items created for this specific study lack the extensive work that foregoes validated questionnaires.

Sixth, the results of the ordinal regression models should be interpreted with caution, particularly in cases when the assumption of proportional odds was not fulfilled. This suggests that the relationship between predictors and the outcome variable may differ across categories and thus future studies could expand on these findings by exploring more flexible modeling approaches.

Seventh, we must acknowledge that our study was observational and exploratory in nature, aiming to assess acceptability and usability constructs without preestablished hypotheses, which warrants some considerations. An important consequence of the observational design is the lack of randomization. As a result, there is potential for selection bias and confounding factors that could influence the results. Concerning the exploratory nature: while this approach provides valuable insights, especially at early stages of development, confirmatory research is needed to test specific predictions and establish causal relationships. This is particularly relevant for the potential between-group differences on how VR exposure relates to cybersickness severity.

Finally, regarding future studies, it is important to highlight that clinician perspectives can form an important complement to patients’ user feedback. This dual approach provides insights that might not be accessible through the patient perspective alone, such as the feasibility and practicality of integrating VR solutions into clinical workflows [70,71]. As such, future studies could consider involving other user groups, and extend the consideration to implementation, feasibility, and cost-effectiveness of VR-based solutions to enable a more comprehensive evaluation.

In summary, despite the limitations and the need for further research, this study is the first to assess the acceptability, usability, and cybersickness levels of a novel VR system incorporating multimodal data for depression symptom assessment. The findings support the potential of VR as a tool for mental health screening, assessment (when used as a supplementary tool), and treatment. While the system’s acceptability was generally favorable, areas for improvement were identified, particularly in enhancing users' self-efficacy during interactions. Usability ratings were satisfactory and cybersickness levels, on average, were comparable to other head-mounted displays. Notably, this study is the first to report elevated cybersickness levels among individuals with DSs, raising important theoretical and methodological questions for future research.

### Conclusions

There is a clear need to enhance mental health diagnosis, potentially by incorporating a broader range of variables. This study explored a novel approach to increase the ecological validity of depression assessments and found that, while VR technologies are generally acceptable as a supplementary tool, they are not seen as a replacement for routine mental health evaluations. The study, which tested the initial version of a novel VR system, revealed that the majority of participants found the acceptability and usability satisfactory, despite experiencing considerable levels of cybersickness—which was found to be negatively associated with both constructs. Notably, the results highlighted previously unreported differences in cybersickness severity between individuals with DSs and HCs, warranting replication and further investigation. While the call for enhanced reliability in mental health assessments is well-founded, and novel approaches are under investigation for their efficacy and accuracy, greater emphasis must be placed on evaluating acceptability and usability. Ensuring a safe, acceptable, and user-friendly approach is essential for the successful implementation of these technologies.

## Appendix

Multimedia Appendix 1 Questionnaire on acceptability based on the Theoretical Framework of Acceptability.

Multimedia Appendix 2 Script used for the analysis and visualization, including its output.

## Abbreviations

DASS: Depression Anxiety Stress Scale
DS: depressive symptom
DSM: Diagnostic and Statistical Manual of Mental Disorders
HC: healthy control
PANAS-SF: Positive and Negative Affect Schedule
PHQ-9: Patient Health Questionnaire-9
SSQ: Simulator Sickness Questionnaire
SUS: System Usability Scale
TFA: theoretical framework of acceptability
VR: virtual reality

## Data Availability

Access to the raw data will be considered on a case-by-case basis upon request. Interested parties may contact the corresponding author.
