# Supplementary material for: Acceptability, Usability, and Insights Into Cybersickness Levels of a Novel Virtual Reality Environment for the Evaluation of Depressive Symptoms: Exploratory Observational Study
Source: JMIR Form Res. 2025 Apr 16;9:e68132. doi: 10.2196/68132 (PMC12044318; doi:10.2196/68132)
Supplement: Multimedia Appendix 1 [file formative_v9i1e68132_app1.docx]

**Supplementary Material 1.**

Questionnaire on acceptability based on the Theoretical Framework of Acceptability [13,19].

The questionnaire was administered in Italian due to the site of recruitment being the University of Padua, Italy.

| **Construct** | **Item** | **Italian translation** |
| --- | --- | --- |
| **Acceptability of the concept** | Please evaluate the following statements after reading the definition of mental health screening, diagnosis and treatment.    **Screening** is used for the early identification of individuals at potentially high risk for a specific condition or disorder; intended for asymptomatic (showing no or disguised symptoms) people.  **Diagnosis** refers to the process of identifying a disorder from its signs and symptoms; thus, intended for those showing symptoms.  **Treatment** refers to medical care given to a patient for a disorder.    Screening for mental disorders via Virtual Reality is acceptable to me.   1. Strongly disagree 2. Disagree 3. No opinion 4. Agree 5. Strongly agree     Diagnosing for mental disorders via Virtual Reality is acceptable to me.   1. Strongly disagree 2. Disagree 3. No opinion 4. Agree 5. Strongly agree   Treatment for mental disorders via Virtual Reality is acceptable to me.   1. Strongly disagree 2. Disagree 3. No opinion 4. Agree 5. Strongly agree | Le chiediamo di valutare le seguenti affermazioni dopo aver letto la definizione di screening, diagnosi e trattamento dei disturbi mentali.  Lo **screening** è utilizzato per l'identificazione precoce di individui a rischio potenzialmente elevato per una specifica patologia o disturbo; è destinato a persone asintomatiche (che non mostrano sintomi o i cui sintomi non sono evidenti).  La **diagnosi** si riferisce al processo di identificazione di un disturbo in base ai suoi segni e sintomi; si rivolge quindi a coloro che presentano sintomi.  Il **trattamento** si riferisce alle cure mediche prestate a un paziente per un disturbo.  Ritengo che sia accettabili condurre lo screening per i disturbi mentali attraverso la realtà virtuale.   1. Fortemente in disaccordo 2. In disaccordo 3. Nessuna opinione 4. D’accordo 5. Fortemente d’accordo   Ritengo che sia accettabile formulare una diagnosi di disturbo mentale attraverso la realtà virtuale.   1. Fortemente in disaccordo 2. In disaccordo 3. Nessuna opinione 4. D’accordo 5. Fortemente d’accordo   Ritengo accettabile l’uso della realtà virtuale per il trattamento dei disturbi mentale.   1. Fortemente in disaccordo 2. In disaccordo 3. Nessuna opinione 4. D’accordo 5. Fortemente d’accordo |
| **Acceptability in practice** | I would be willing to engage with the EXPERIENCE system to inform my diagnosis made by a mental health professional.   1. Strongly disagree 2. Disagree 3. No opinion 4. Agree 5. Strongly agree   I would be willing to engage with the EXPERIENCE system to receive a diagnosis on my mental health / depressive state without any extra input from a mental health professional.   1. Strongly disagree 2. Disagree 3. No opinion 4. Agree 5. Strongly agree | Sarei disposto ad utilizzare il sistema EXPERIENCE per offrire informazioni utili per formulare una diagnosi clinica da un professionista della salute mentale.   1. Fortemente in disaccordo 2. In disaccordo 3. Nessuna opinione 4. D’accordo 5. Fortemente d’accordo   Sarei disposto ad utilizzare il sistema EXPERIENCE per ricevere una diagnosi sulla mia salute mentale o sul mio stato depressivo senza l'ausilio di un professionista della salute mentale.   1. Fortemente in disaccordo 2. In disaccordo 3. Nessuna opinione 4. D’accordo 5. Fortemente d’accordo |
| **General acceptability** | How acceptable was the EXPERIENCE system for you?   1. Completely unacceptable 2. Unacceptable 3. No opinion 4. Acceptable 5. Completely acceptable | L’esperienza con il sistema EXPERIENCE è stata per lei   1. Completamente inaccettabile 2. Inaccettabile 3. Nessuna opinione 4. Accettabile 5. Completamente accettabile |
| **Affective attitudes** | Did you like or dislike the EXPERIENCE system?   1. Strongly dislike 2. Dislike 3. No opinion 4. Like 5. Strongly like   How comfortable did you feel while engaging with the EXPERIENCE system?   1. Very uncomfortable 2. Uncomfortable 3. No opinion 4. Comfortable 5. Very comfortable | Il sistema EXPERIENCE ti è piaciuto oppure no?   1. Non mi è piaciuto per niente 2. Non mi è piaciuto 3. Nessuna opinione 4. Mi è piaciuto 5. Mi è piaciuto molto   Quanto ti sentivi a tuo agio durante l’interazione col sistema EXPERIENCE?   1. Molto a disagio 2. A disagio 3. Nessuna opinione 4. A mio agio 5. Molto a mio agio |
| **Perceived effectiveness** | The EXPERIENCE system has improved my mental state.   1. Strongly disagree 2. Disagree 3. No opinion 4. Agree 5. Strongly agree | Il sistema EXPERIENCE ha migliorato il mio stato mentale.   1. Fortemente in disaccordo 2. In disaccordo 3. Nessuna opinione 4. D’accordo 5. Fortemente d’accordo |
| **Intervention coherence** | It is clear to me how the EXPERIENCE system will help to diagnose depressive disorders.   1. Strongly disagree 2. Disagree 3. No opinion 4. Agree 5. Strongly agree | Mi è chiaro come il sistema EXPERIENCE potrebbe aiutare a diagnosticare i disturbi depressivi.   1. Fortemente in disaccordo 2. In disaccordo 3. Nessuna opinione 4. D’accordo 5. Fortemente d’accordo |
| **Burden**  *(reversed)* | How much effort did it take to engage with the EXPERIENCE system?   1. No effort at all 2. A little effort 3. No opinion 4. A lot of effort 5. Huge effort | Quanto sforzo ha richiesto l'utilizzo del sistema EXPERIENCE?   1. Nessuno sforzo 2. Un po’ di sforzo 3. Nessuna opinione 4. Molto sforzo 5. Un enorme sforzo |
| **Ethicality**  *(reversed)* | There are moral or ethical consequences to engaging with the EXPERIENCE system.   1. 1 – Strongly disagree 2. 2 – Disagree 3. 3 – No opinion 4. 4 – Agree 5. 5 – Strongly agree | L'utilizzo del sistema EXPERIENCE ha conseguenze morali o etiche.   1. Fortemente in disaccordo 2. In disaccordo 3. Nessuna opinione 4. D’accordo 5. Fortemente d’accordo |
| **Opportunity cost**  *(reversed)* | I worry that engaging with the EXPERIENCE system will lead to loss of other diagnostic opportunities.   1. Strongly disagree 2. Disagree 3. No opinion 4. Agree 5. Strongly agree | Mi preoccupa che l’utlizzo del sistema EXPERIENCE potrebbe causare la perdita di altre alternative diagnostiche.   1. Fortemente in disaccordo 2. In disaccordo 3. Nessuna opinione 4. D’accordo 5. Fortemente d’accordo |
| **Self-efficacy**  *(Taken from the System Usability Scale - Item 9.)* | I felt very confident using the system.   1. Strongly disagree 2. Disagree 3. No opinion 4. Agree 5. Strongly agree | Ho avuto molta confidenza con il sistema durante l'uso.   1. Fortemente in disaccordo 2. In disaccordo 3. Nessuna opinione 4. D’accordo 5. Fortemente d’accordo |
| **Perceived usefulness** | How useful do you think the EXPERIENCE system was?   1. Not at all useful 2. Not useful 3. No opinion 4. Useful 5. Very useful     What do you think the EXPERIENCE system was useful for?  [ ] Better understanding of my mental state  [ ] My mental state improved [ ] Other: | Quanto ritiene sia stato utile il sistema EXPERIENCE?   1. Completamente inutile 2. Inutile 3. Nessuna opinione 4. Utile 5. Molto utile   Per cosa pensa sia stato utile il sistema EXPERIENCE?  [ ] Una migliore comprensione del mio stato mentale  [ ] un miglioramento del mio stato mentale  [ ] Altro: |
