# Supplementary material for: Acceptability, Usability, and Insights Into Cybersickness Levels of a Novel Virtual Reality Environment for the Evaluation of Depressive Symptoms: Exploratory Observational Study
Source: JMIR Form Res. 2025 Apr 16;9:e68132. doi: 10.2196/68132 (PMC12044318; doi:10.2196/68132)
Supplement: Multimedia Appendix 2 [file formative_v9i1e68132_app2.pdf]

Supplementary Material 2

Sara Sutori

Fundamentals

Get the version of your RStudio

```
if (interactive()) {
  # Only run this code when in an interactive session (e.g., RStudio)
  print(RStudio.Version())
}
```

Clear things and set random seed

```
rm(list = ls())
graphics.off()
cat("\014")
```

```r
set.seed(123)
```

Download libraries if necessary

Load libraries

```r
library("dplyr")        # For data management
library("tidyverse")    # For data management
library("readxl")       # For importing data from excel
library("ggplot2")      # For visualization purposes
library("vioplot")      # For visualization purposes
library("ordinal")      # For dealing with ordinal outcomes
library("brant")        # For testing the requirement of proportional odds
library("MASS")
library("psych")
library("rstatix")
library("coin")
library("ggeffects")    # For the purpose of obtaining predicted values from regression models
library("VGAM")         # For employing the Partial Proportional Odds Models
library("rmarkdown")    # For the use of rmarkdown
library("effectsize")  # Calculate effect sizes
library("rmarkdown")    # Save file to pdf
```

Import dataset

Direct to the folder on the computer

Load data

```r
# Import dataset
d = read_excel('EXP_master_dataset_2025March.xlsx', na = "NA")
```

Define variable types

```r
d$SSQ_Total <- as.numeric(d$SSQ_Total)
d$SUS_Total <- as.numeric(d$SUS_Total)
d$Nausea <- as.numeric(d$Nausea)
d$Oculomotor <- as.numeric(d$Oculomotor)
d$Disorientation <- as.numeric(d$Disorientation)
d$Screening_disorder   <- as.numeric(d$Screening_disorder)
d$Diagnosing_disorder   <- as.numeric(d$Diagnosing_disorder)
d$Treatment_disorder   <- as.numeric(d$Treatment_disorder)
d$Treatment_depression   <- as.numeric(d$Treatment_depression)
d$Willing_diagnosis_HP   <- as.numeric(d$Willing_diagnosis_HP)
d$Willing_diagnosis_NHP   <- as.numeric(d$Willing_diagnosis_NHP)
d$Acceptable   <- as.numeric(d$Acceptable)
```

```r
d$Like_dislike   <- as.numeric(d$Like_dislike)
d$Comfortable   <- as.numeric(d$Comfortable)
d$Improved_mental_state   <- as.numeric(d$Improved_mental_state)
d$Clarity_diagnosis   <- as.numeric(d$Clarity_diagnosis)
d$Confident_SUS9   <- as.numeric(d$Confident_SUS9)
d$Effort   <- as.numeric(d$Effort)
d$Effort_rev   <- as.numeric(d$Effort_rev)
d$Moral_ethical   <- as.numeric(d$Moral_ethical)
d$Moral_ethical_rev   <- as.numeric(d$Moral_ethical_rev)
d$Opportunity_cost   <- as.numeric(d$Opportunity_cost)
d$Opportunity_cost_rev   <- as.numeric(d$Opportunity_cost_rev)
d$Usefulness   <- as.numeric(d$Usefulness)
d$Nausea   <- as.numeric(d$Nausea)
d$Oculomotor   <- as.numeric(d$Oculomotor)
d$Disorientation   <- as.numeric(d$Disorientation)
```

Brief look at the data

```r
# Get the volume of data
length(d$ID)
```

```
## [1] 100
```

```r
nrow(d)
```

```
## [1] 100
```

```r
# Get a summary of the data
summary(d)
```

```
##       ID                 Date                 Gender    Hand_dominance
##  Length:100         Length:100         Min.   :1.0   Min.   :1.00
##  Class :character   Class :character   1st Qu.:2.0   1st Qu.:2.00
##  Mode  :character   Mode  :character   Median :2.0   Median :2.00
##                                        Mean   :1.8   Mean   :1.97
##                                        3rd Qu.:2.0   3rd Qu.:2.00
##                                        Max.   :2.0   Max.   :2.00
##
##       Age          Education         Weight          Height
##  Min.   :19.00   Min.   :12.00   Min.   :45.00   Min.   :1.550
##  1st Qu.:22.00   1st Qu.:16.00   1st Qu.:55.00   1st Qu.:1.607
##  Median :23.00   Median :17.00   Median :60.00   Median :1.655
##  Mean   :23.17   Mean   :16.34   Mean   :61.17   Mean   :1.668
##  3rd Qu.:24.00   3rd Qu.:17.00   3rd Qu.:65.25   3rd Qu.:1.712
##  Max.   :28.00   Max.   :18.00   Max.   :92.00   Max.   :1.820
##
##       BMI        Sight_correction Color_blindness Sleep_general
##  Min.   :17.30   Min.   :1.00     Min.   :1       Min.   : 5.000
##  1st Qu.:20.03   1st Qu.:1.00     1st Qu.:1       1st Qu.: 6.500
##  Median :21.50   Median :2.00     Median :1       Median : 7.000
```

```
##  Mean   :21.93   Mean   :1.86     Mean   :1       Mean   : 7.155
##  3rd Qu.:23.48   3rd Qu.:3.00     3rd Qu.:1       3rd Qu.: 8.000
##  Max.   :28.12   Max.   :3.00     Max.   :1       Max.   :10.500
##
##  Sleep_previous   Habitual_alcohol Alcohol_yesterday  Alcohol_week
##  Min.   : 3.000   Min.   :1.00     Min.   :0.0000    Min.   : 0
##  1st Qu.: 6.000   1st Qu.:1.00     1st Qu.:0.0000    1st Qu.: 0
##  Median : 7.000   Median :2.00     Median :0.0000    Median : 2
##  Mean   : 6.975   Mean   :1.73     Mean   :0.4639    Mean   : 2
##  3rd Qu.: 8.000   3rd Qu.:2.00     3rd Qu.:1.0000    3rd Qu.: 3
##  Max.   :10.000   Max.   :2.00     Max.   :4.0000    Max.   :15
##                                    NA's   :3
##     Smoking     Cigarettes_day  Psychoactive_drugs
##  Min.   :1.00   Min.   : 0.00   Min.   :1.00
##  1st Qu.:1.00   1st Qu.: 0.00   1st Qu.:1.00
##  Median :1.00   Median : 0.00   Median :1.00
##  Mean   :1.39   Mean   : 2.43   Mean   :1.15
##  3rd Qu.:2.00   3rd Qu.: 4.00   3rd Qu.:1.00
##  Max.   :2.00   Max.   :15.00   Max.   :2.00
##
##  Psychoactive_drugs_day_before Cardiovascular_migraine Pharmacological_therapy
##  Min.   :1                     Min.   :1.00            Min.   :1.00
##  1st Qu.:1                     1st Qu.:1.00            1st Qu.:1.00
##  Median :1                     Median :1.00            Median :1.00
##  Mean   :1                     Mean   :1.05            Mean   :1.16
##  3rd Qu.:1                     3rd Qu.:1.00            3rd Qu.:1.00
##  Max.   :1                     Max.   :3.00            Max.   :5.00
##
##  PANAS_POS_PRE   PANAS_NEG_PRE      PHQ_Tot         Group
##  Min.   : 0.00   Min.   : 0.00   Min.   : 0.00   Length:100
##  1st Qu.: 9.00   1st Qu.: 0.00   1st Qu.: 4.00   Class :character
##  Median :11.50   Median : 2.00   Median : 7.00   Mode  :character
##  Mean   :11.28   Mean   : 2.56   Mean   : 7.27
##  3rd Qu.:14.00   3rd Qu.: 4.00   3rd Qu.:11.00
##  Max.   :17.00   Max.   :11.00   Max.   :15.00
##
##      DASS_S          DASS_A       DASS_D        CEI_Total     CEI_Stretching
##  Min.   : 0.00   Min.   : 0   Min.   : 0.00   Min.   :13.00   Min.   : 6.00
##  1st Qu.: 6.00   1st Qu.: 0   1st Qu.: 2.00   1st Qu.:26.00   1st Qu.:14.75
##  Median :12.00   Median : 4   Median : 6.00   Median :31.50   Median :17.00
##  Mean   :12.74   Mean   : 6   Mean   : 9.36   Mean   :31.09   Mean   :17.06
##  3rd Qu.:18.00   3rd Qu.:10   3rd Qu.:14.00   3rd Qu.:36.00   3rd Qu.:20.00
##  Max.   :36.00   Max.   :28   Max.   :30.00   Max.   :45.00   Max.   :24.00
##
##  CEI_Embracing   PANAS_POS_POST  PANAS_NEG_POST      Nausea
##  Min.   : 6.00   Min.   : 0.00   Min.   : 0.00   Min.   :  0.00
##  1st Qu.:11.00   1st Qu.: 7.00   1st Qu.: 0.00   1st Qu.:  9.54
##  Median :14.00   Median :11.00   Median : 1.00   Median : 19.08
##  Mean   :14.03   Mean   :10.34   Mean   : 2.46   Mean   : 21.59
##  3rd Qu.:17.00   3rd Qu.:14.00   3rd Qu.: 4.00   3rd Qu.: 28.62
##  Max.   :21.00   Max.   :20.00   Max.   :11.00   Max.   :114.48
##                                                  NA's   :1
##    Oculomotor    Disorientation     SSQ_Total        SUS_Total
##  Min.   : 0.00   Min.   :  0.00   Min.   :  0.00   Min.   :37.50
```

```
##  1st Qu.: 7.58   1st Qu.:  0.00   1st Qu.:  7.48   1st Qu.:62.50
##  Median :22.74   Median : 13.92   Median : 22.44   Median :70.00
##  Mean   :26.72   Mean   : 32.90   Mean   : 30.49   Mean   :69.02
##  3rd Qu.:37.90   3rd Qu.: 41.76   3rd Qu.: 44.88   3rd Qu.:77.50
##  Max.   :98.54   Max.   :194.88   Max.   :119.68   Max.   :97.50
##  NA's   :1       NA's   :1        NA's   :1        NA's   :1
##  Screening_disorder Diagnosing_disorder Treatment_disorder Treatment_depression
##  Min.   :1.000      Min.   :1.000       Min.   :1.000      Min.   :1.00
##  1st Qu.:4.000      1st Qu.:2.000       1st Qu.:3.000      1st Qu.:3.00
##  Median :4.000      Median :3.000       Median :4.000      Median :4.00
##  Mean   :3.833      Mean   :2.771       Mean   :3.774      Mean   :3.75
##  3rd Qu.:4.000      3rd Qu.:4.000       3rd Qu.:4.000      3rd Qu.:4.00
##  Max.   :5.000      Max.   :5.000       Max.   :5.000      Max.   :5.00
##  NA's   :16         NA's   :17          NA's   :16         NA's   :16
##  Willing_diagnosis_HP Willing_diagnosis_NHP   Acceptable     Like_dislike
##  Min.   :1.000        Min.   :1             Min.   :2.000   Min.   :2.000
##  1st Qu.:3.500        1st Qu.:1             1st Qu.:4.000   1st Qu.:4.000
##  Median :4.000        Median :2             Median :4.000   Median :4.000
##  Mean   :3.843        Mean   :2             Mean   :4.369   Mean   :4.181
##  3rd Qu.:4.500        3rd Qu.:3             3rd Qu.:5.000   3rd Qu.:5.000
##  Max.   :5.000        Max.   :5             Max.   :5.000   Max.   :5.000
##  NA's   :17           NA's   :16            NA's   :16      NA's   :17
##   Comfortable    Improved_mental_state Clarity_diagnosis Confident_SUS9
##  Min.   :2.000   Min.   :1.000         Min.   :1.000     Min.   :1.000
##  1st Qu.:3.000   1st Qu.:2.000         1st Qu.:3.000     1st Qu.:2.000
##  Median :4.000   Median :3.000         Median :4.000     Median :3.000
##  Mean   :3.571   Mean   :2.869         Mean   :3.702     Mean   :2.798
##  3rd Qu.:4.000   3rd Qu.:3.000         3rd Qu.:4.000     3rd Qu.:3.000
##  Max.   :5.000   Max.   :5.000         Max.   :5.000     Max.   :5.000
##  NA's   :16      NA's   :16            NA's   :16        NA's   :1
##      Effort       Effort_rev   Moral_ethical   Moral_ethical_rev
##  Min.   :1.00   Min.   :2.00   Min.   :1.000   Min.   :1.000
##  1st Qu.:2.00   1st Qu.:4.00   1st Qu.:2.000   1st Qu.:3.000
##  Median :2.00   Median :4.00   Median :2.000   Median :4.000
##  Mean   :2.19   Mean   :3.81   Mean   :2.393   Mean   :3.619
##  3rd Qu.:2.00   3rd Qu.:4.00   3rd Qu.:3.000   3rd Qu.:4.000
##  Max.   :4.00   Max.   :5.00   Max.   :5.000   Max.   :5.000
##  NA's   :16     NA's   :16     NA's   :16      NA's   :16
##  Opportunity_cost Opportunity_cost_rev   Usefulness      Useful_for
##  Min.   :1.000    Min.   :1.000        Min.   :2.000   Min.   : 0.000
##  1st Qu.:2.000    1st Qu.:2.000        1st Qu.:3.000   1st Qu.: 1.000
##  Median :2.000    Median :4.000        Median :4.000   Median : 1.000
##  Mean   :2.786    Mean   :3.202        Mean   :3.774   Mean   : 2.207
##  3rd Qu.:4.000    3rd Qu.:4.000        3rd Qu.:4.000   3rd Qu.: 3.000
##  Max.   :5.000    Max.   :5.000        Max.   :5.000   Max.   :12.000
##  NA's   :16       NA's   :16           NA's   :16      NA's   :18
##  Accep_other_IT     Accep_other_EN     TimeEnvironment
##  Length:100         Length:100         Min.   :1162
##  Class :character   Class :character   1st Qu.:1609
##  Mode  :character   Mode  :character   Median :1803
##                                        Mean   :1886
##                                        3rd Qu.:2076
##                                        Max.   :3031
##                                        NA's   :1
```

Descriptives

Sample characteristics

```r
# Create a function to combine mean and SD into a single string
mean_sd <- function(x) {
  mean_value <- mean(x, na.rm = TRUE)
  sd_value <- sd(x, na.rm = TRUE)
  return(paste0(round(mean_value, 2), " (", round(sd_value, 2), ")"))
}

# Apply the function to each variable, grouped by the 'Group' variable (control vs. depressed)
summary_table <- d %>%
  group_by(Group) %>%
  summarise(
    Age = mean_sd(Age),
    Education = mean_sd(Education),
    Height = mean_sd(Height),
    Weight = mean_sd(Weight),
    BMI = mean_sd(BMI),
    Sleep_general = mean_sd(Sleep_general),
    PHQ_Tot = mean_sd(PHQ_Tot),
    DASS_D = mean_sd(DASS_D),
    DASS_A = mean_sd(DASS_A),
    DASS_S = mean_sd(DASS_S),
    PANAS_POS_PRE = mean_sd(PANAS_POS_PRE),
    PANAS_NEG_PRE = mean_sd(PANAS_NEG_PRE),
    PANAS_POS_POST = mean_sd(PANAS_POS_POST),
    PANAS_NEG_POST = mean_sd(PANAS_NEG_POST)
  ) %>%
  ungroup() %>%
  # Transpose the table for a more compact format
  tidyr::pivot_longer(cols = -Group, names_to = "Variable", values_to = "Mean (SD)") %>%
  tidyr::pivot_wider(names_from = Group, values_from = `Mean (SD)`)

# Display the summary table
print(summary_table)
```

```
## # A tibble: 14 x 3
##    Variable      Control      Depressed
##    <chr>         <chr>        <chr>
##  1 Age           23.34 (1.52) 23 (1.86)
##  2 Education     16.58 (0.78) 16.1 (1.4)
##  3 Height        1.66 (0.07)  1.67 (0.07)
##  4 Weight        59.63 (7.85) 62.72 (10.6)
##  5 BMI           21.5 (2.06)  22.37 (3)
##  6 Sleep_general 7.31 (0.88)  7 (0.98)
##  7 PHQ_Tot       3.24 (1.48)  11.3 (1.92)
##  8 DASS_D        3.52 (3.44)  15.2 (7.4)
##  9 DASS_A        2.44 (2.89)  9.56 (6.62)
## 10 DASS_S        7.72 (5.08)  17.76 (6.73)
## 11 PANAS_POS_PRE 12.18 (2.76) 10.38 (3.53)
```

```
## 12 PANAS_NEG_PRE  1.88 (2.22)  3.24 (2.98)
## 13 PANAS_POS_POST 11.8 (3.61)  8.88 (4.36)
## 14 PANAS_NEG_POST 1.72 (2.24)  3.2 (3.25)
```

```r
## Between group differences
# Create a function to perform Mann-Whitney U test and calculate effect size
perform_mann_whitney <- function(variable, group_var) {
  test_result <- wilcox.test(variable ~ group_var, exact = FALSE, na.rm = TRUE)
  effect_size <- wilcox_effsize(data.frame(variable = variable, group_var = group_var), variable ~ grou

  p_value <- test_result$p.value
  effect_size_r <- effect_size$effsize

  return(list(p_value = p_value, effect_size = effect_size_r))
}

# List of variables to test
variables <- c("Age", "Education", "Height", "Weight", "BMI", "Sleep_general", "PHQ_Tot", "DASS_D", "DAS
               "PANAS_POS_PRE", "PANAS_NEG_PRE", "PANAS_POS_POST", "PANAS_NEG_POST")

# Create an empty data frame to store the results
results_desc <- data.frame(Variable = character(),
                      P_Value = numeric(),
                      Effect_Size = numeric(),
                      stringsAsFactors = FALSE)

# Loop through each variable and perform the test
for (var in variables) {
  test_results <- perform_mann_whitney(d[[var]], d$Group)

  results_desc <- rbind(results_desc, data.frame(
    Variable = var,
    P_Value = round(test_results$p_value, 3),
    Effect_Size = round(test_results$effect_size, 2)
  ))
}

# Print the results table
print(results_desc)
```

```
##                           Variable P_Value Effect_Size
## Effect size (r)                Age   0.200        0.13
## Effect size (r)1         Education   0.150        0.14
## Effect size (r)2            Height   0.507        0.07
## Effect size (r)3            Weight   0.226        0.12
## Effect size (r)4               BMI   0.206        0.13
## Effect size (r)5     Sleep_general   0.050        0.20
## Effect size (r)6           PHQ_Tot   0.000        0.87
## Effect size (r)7            DASS_D   0.000        0.77
## Effect size (r)8            DASS_A   0.000        0.61
## Effect size (r)9            DASS_S   0.000        0.66
## Effect size (r)10    PANAS_POS_PRE   0.008        0.27
## Effect size (r)11    PANAS_NEG_PRE   0.017        0.24
## Effect size (r)12 PANAS_POS_POST   0.000        0.36
```

```
## Effect size (r)13 PANAS_NEG_POST   0.016        0.24
```

```r
# Combine above tables and create one descriptive table
# Add significance stars to p-values
results_desc <- results_desc %>%
  mutate(Significance = case_when(
    P_Value < 0.001 ~ "***",
    P_Value < 0.01  ~ "**",
    P_Value < 0.05  ~ "*",
    TRUE            ~ ""
  ))

# Combine p-values and effect sizes with stars
results_desc <- results_desc %>%
  mutate(R_Value = paste0(Effect_Size, Significance))

# Merge the two tables
final_table <- summary_table %>%
  left_join(results_desc %>% select(Variable, R_Value), by = "Variable") %>%
  rename(
    Control = `Control`,
    Depressed = `Depressed`,
    `R (p)` = R_Value
  )

# Display the final table
print(final_table)
```

```
## # A tibble: 14 x 4
##    Variable       Control      Depressed    'R (p)'
##    <chr>          <chr>        <chr>        <chr>
##  1 Age            23.34 (1.52) 23 (1.86)    0.13
##  2 Education      16.58 (0.78) 16.1 (1.4)   0.14
##  3 Height         1.66 (0.07)  1.67 (0.07)  0.07
##  4 Weight         59.63 (7.85) 62.72 (10.6) 0.12
##  5 BMI            21.5 (2.06)  22.37 (3)    0.13
##  6 Sleep_general  7.31 (0.88)  7 (0.98)     0.2
##  7 PHQ_Tot        3.24 (1.48)  11.3 (1.92)  0.87***
##  8 DASS_D         3.52 (3.44)  15.2 (7.4)   0.77***
##  9 DASS_A         2.44 (2.89)  9.56 (6.62)  0.61***
## 10 DASS_S         7.72 (5.08)  17.76 (6.73) 0.66***
## 11 PANAS_POS_PRE  12.18 (2.76) 10.38 (3.53) 0.27**
## 12 PANAS_NEG_PRE  1.88 (2.22)  3.24 (2.98)  0.24*
## 13 PANAS_POS_POST 11.8 (3.61)  8.88 (4.36)  0.36***
## 14 PANAS_NEG_POST 1.72 (2.24)  3.2 (3.25)   0.24*
```

Visualization

Figure 3. Distribution of participants' scores on the Patient Health Questionnaire (PHQ-9) assessing current depressive symptom severity.

```r
# Histogram on PHQ-9 score distribution
PHQ_distibution_full <-
  ggplot(d, aes(x = PHQ_Tot, fill = Group)) +
  geom_histogram(binwidth = 1, color = "#e9ecef", alpha = 0.9) +
  xlab("Depressive symptom severity (PHQ-9 score)") +
  ylab(bquote("Number of participants")) +
  scale_fill_manual(
    values = c("grey", "#33cc33"),
    labels = c("Healthy control", "Depressive symptoms")
  ) +
  theme(text = element_text(size = 14)) +
  scale_x_continuous(
    limits = c(-1, 27),  # Set x-axis limits from 0 to 27
    breaks = seq(0, 27, by = 5)
  )

print(PHQ_distibution_full)
```

```
## Warning: Removed 4 rows containing missing values or values outside the scale range
## (`geom_bar()`).
```

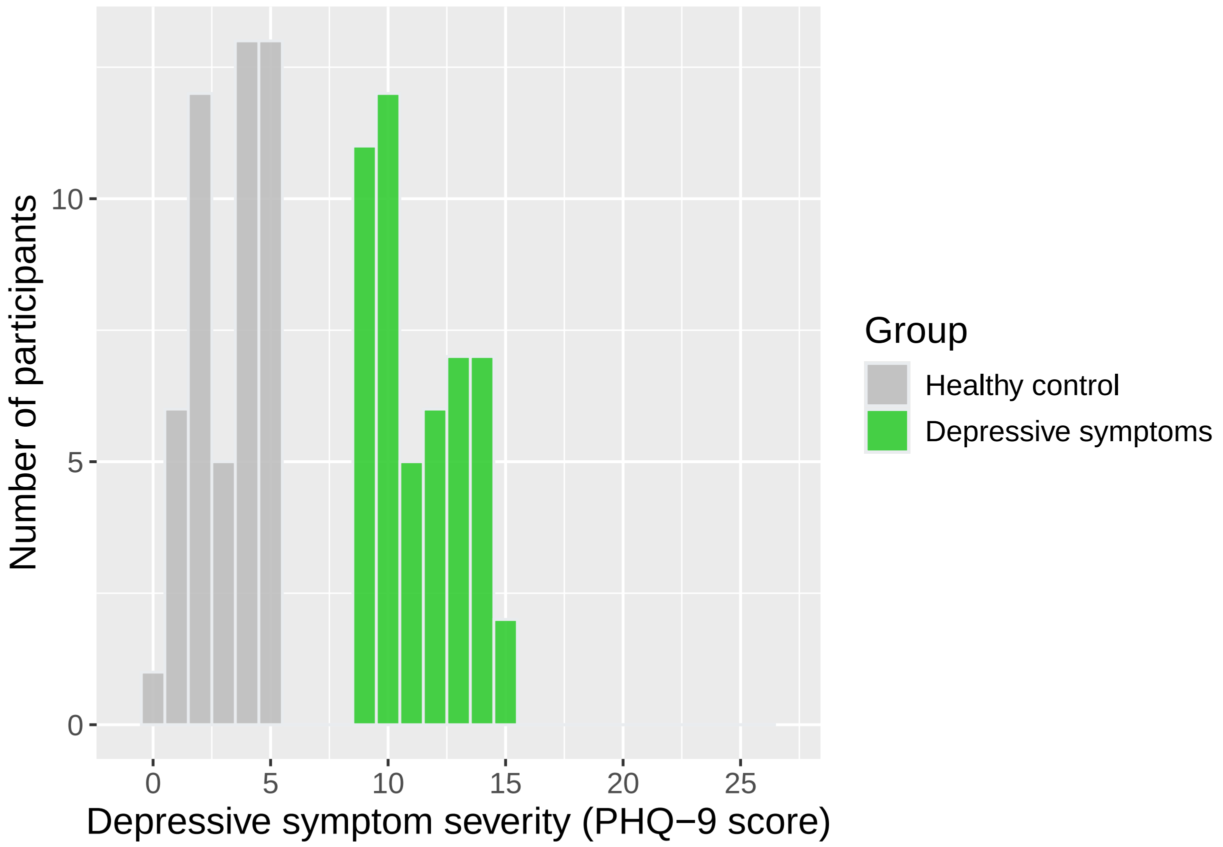


```r
# Save figure in high resolution
par(mfrow=c(1,1))
png("PHQ-9_distribution.png", units="px", width=2400, height=1680, res=300)
print(PHQ_distibution_full)
```

```
## Warning: Removed 4 rows containing missing values or values outside the scale range
## (`geom_bar()`).
```

```r
dev.off()
```

```
## pdf
##   2
```

Acceptability of the concept

Descriptives

In relation to mental disorders

Screening

```r
mean(d$Screening_disorder, na.rm = T)
```

```
## [1] 3.833333
```

```r
sd(d$Screening_disorder, na.rm = T)
```

```
## [1] 0.9673381
```

```r
sum(d$Screening_disorder <3 , na.rm = T)         # Number of those finding the system unacceptable (scor
```

```
## [1] 11
```

```r
sum(d$Screening_disorder!="", na.rm = T)         # Total number of values
```

```
## [1] 84
```

```r
sum(d$Screening_disorder >3 , na.rm = T) / sum(d$Screening_disorder!="", na.rm = T) * 100 #Percentage o
```

```
## [1] 77.38095
```

```r
table(d$Screening_disorder)
```

```
##
##  1  2  3  4  5
##  2  9  8 47 18
```

Diagnosis

```r
mean(d$Diagnosing_disorder, na.rm = T)
```

```
## [1] 2.771084
```

```r
sd(d$Diagnosing_disorder, na.rm = T)
```

```
## [1] 1.232935
```

```r
sum(d$Diagnosing_disorder <3 , na.rm = T)        # Number of those finding the system unacceptable
```

```
## [1] 41
```

```r
sum(d$Diagnosing_disorder!="", na.rm = T)        # Total number of values
```

```
## [1] 83
```

```r
sum(d$Diagnosing_disorder >3 , na.rm = T) / sum(d$Diagnosing_disorder!="", na.rm = T) * 100
```

```
## [1] 33.73494
```

```r
table(d$Diagnosing_disorder)
```

```
##
##  1  2  3  4  5
## 13 28 14 21  7
```

Treatment

```r
# Treatment
mean(d$Treatment_disorder, na.rm = T)
```

```
## [1] 3.77381
```

```r
sd(d$Treatment_disorder, na.rm = T)
```

```
## [1] 0.9982055
```

```r
sum(d$Treatment_disorder <3 , na.rm = T)         # Number of those finding the system unacceptable
```

```
## [1] 11
```

```r
sum(d$Treatment_disorder!="", na.rm = T)         # Total number of values
```

```
## [1] 84
```

```r
sum(d$Treatment_disorder >3 , na.rm = T) / sum(d$Treatment_disorder!="", na.rm = T) * 100
```

```
## [1] 73.80952
```

```r
table(d$Treatment_disorder)
```

```
##
##  1  2  3  4  5
##  3  8 11 45 17
```

In practice for the purpose of diagnosis

As a support tool used by a mental health professional

```r
mean(d$Willing_diagnosis_HP, na.rm = T)
```

```
## [1] 3.843373
```

```r
sd(d$Willing_diagnosis_HP, na.rm = T)
```

```
## [1] 1.029823
```

```r
sum(d$Willing_diagnosis_HP <3 , na.rm = T)      # Number of those finding the system unacceptable
```

```
## [1] 9
```

```r
sum(d$Willing_diagnosis_HP!="", na.rm = T)      # Total number of values
```

```
## [1] 83
```

```r
sum(d$Willing_diagnosis_HP >3 , na.rm = T) / sum(d$Willing_diagnosis_HP!="", na.rm = T) * 100
```

```
## [1] 74.6988
```

As a standalone tool without input from a mental health professional

```r
mean(d$Willing_diagnosis_NHP, na.rm = T)
```

```
## [1] 2
```

```r
sd(d$Willing_diagnosis_NHP, na.rm = T)
```

```
## [1] 1.212386
```

```r
sum(d$Willing_diagnosis_NHP <3 , na.rm = T)     # Number of those finding the system unacceptable
```

```
## [1] 62
```

```r
sum(d$Willing_diagnosis_NHP!="", na.rm = T)     # Total number of values
```

```
## [1] 84
```

```r
sum(d$Willing_diagnosis_NHP >3 , na.rm = T) / sum(d$Willing_diagnosis_NHP!="", na.rm = T) * 100
```

```
## [1] 17.85714
```

Visualization

Figure 5. Participant ratings of Virtual Reality acceptance for the purposes of mental health screening, diagnosis, and treatment.

```r
## Figure on main concepts - stacked bar graph
# Reshape data from wide to long format
d_long <- d %>%
  pivot_longer(cols = c(Screening_disorder, Diagnosing_disorder, Treatment_disorder),
               names_to = "Disorder",
               values_to = "Rating") %>%
  # Rename the variables
  mutate(Disorder = recode(Disorder,
                           "Screening_disorder" = "Screening",
                           "Diagnosing_disorder" = "Diagnosis",
                           "Treatment_disorder" = "Treatment"),
         Rating = ifelse(is.na(Rating), "N/A", Rating))  # Replace NA with "N/A")

# Convert Rating to a factor with reversed levels for stacking
d_long$Rating <- factor(d_long$Rating, levels = c("5", "4", "3", "2", "1","N/A"))

# Define the color palette
color_palette <- c("N/A" = "azure4", "1" = "#ff6666", "2" = "#ff9999",
                   "3" = "lightgrey", "4" = "#66ff66", "5" = "#33cc33")

# Define custom labels for the legend
custom_labels <- c("N/A" = "N/A",
                   "1" = "Strongly disagree",
                   "2" = "Disagree",
                   "3" = "No opinion",
                   "4" = "Agree",
                   "5" = "Strongly agree")

# Create the stacked bar plot with custom colors and legend labels
bar_graph <- ggplot(d_long, aes(x = Disorder, fill = Rating)) +
  geom_bar(position = position_stack(reverse = TRUE)) +
  scale_fill_manual(values = color_palette, labels = custom_labels) +
  labs(
```

```
    title = "Use of VR for the purpose of mental health ... is acceptable to me.", # Graph title
    x = NULL, # Remove x-axis title
    y = "Count", # Y-axis title
    fill = "Rating"
  ) +
  theme_minimal() +
  coord_flip() +  # Ensure bars are horizontal
  theme(
    plot.title = element_text(size = 10, face = "bold", hjust = 0.5), # Title size and alignment
    axis.title.x = element_text(size = 10),  # Axis title size
    axis.title.y = element_text(size = 10),  # Y-axis title size
    axis.text.x = element_text(size = 10),   # Axis text size
    axis.text.y = element_text(size = 10),   # Axis text size
    legend.title = element_text(size = 10),  # Legend title size
    legend.text = element_text(size = 10),   # Legend text size
    legend.key.size = unit(0.3, "lines")     # Smaller legend keys
  )

print(bar_graph)
```

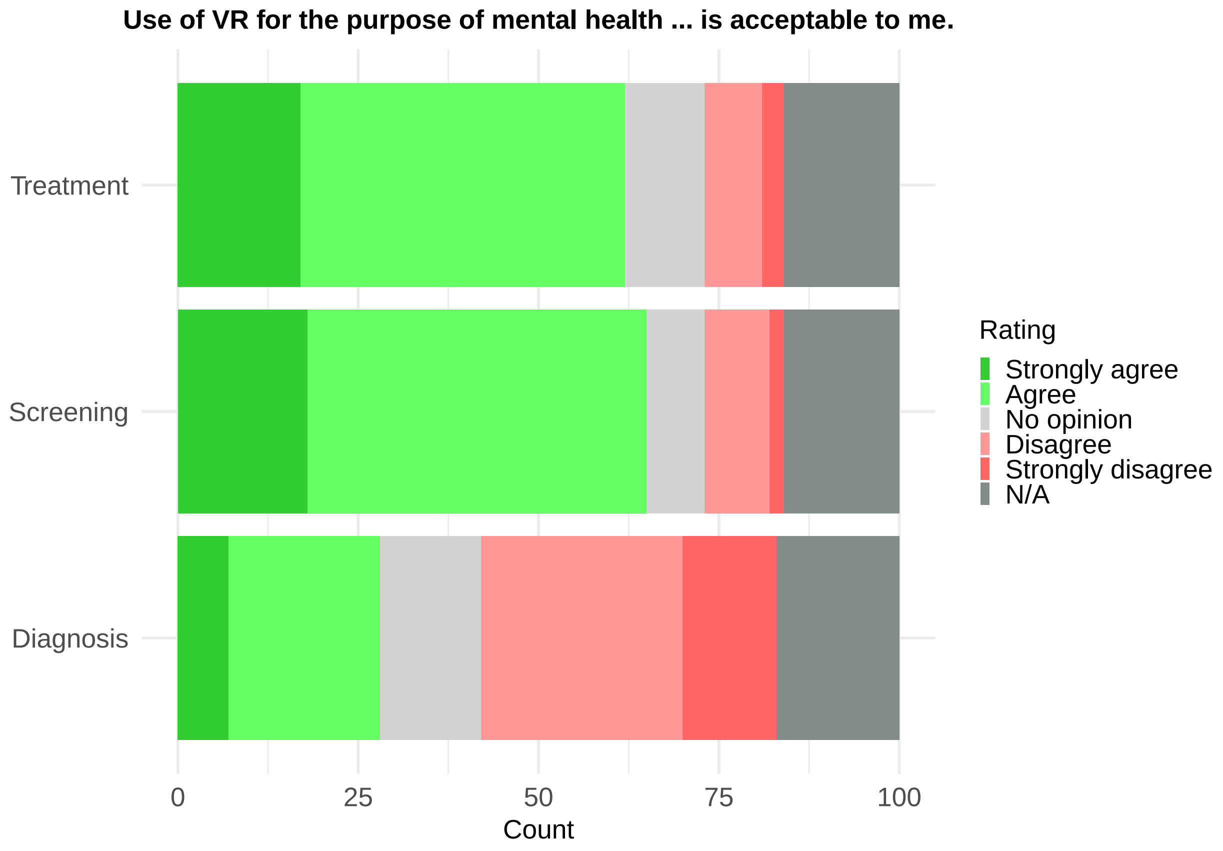


```
# Save in high resolution
png("Bar_Figure.png", units="px", width=2400, height=620, res=300)
print (bar_graph)
dev.off()
```

```
## pdf
```

```
## 	2
```

Figure 6. Participants' subjective ratings of their willingness to engage with the pilot VR system for mental health diagnosis. The support tool complements the assessment of a mental health professional, whereas the standalone tool operates without professional input.

```
## Figure on diagnostic components - stacked bar graph
# Reshape data from wide to long format
d_long <- d %>%
  pivot_longer(cols = c(Willing_diagnosis_HP, Willing_diagnosis_NHP),
               names_to = "Diagnosis",
               values_to = "Rating") %>%
  # Rename the variables
  mutate(Diagnosis = recode(Diagnosis,
                            "Willing_diagnosis_HP" = "Support tool",
                            "Willing_diagnosis_NHP" = "Standalone tool"),
         Rating = ifelse(is.na(Rating), "N/A", Rating))  # Replace NA with "N/A"))

# Convert Rating to a factor with reversed levels for stacking
d_long$Rating <- factor(d_long$Rating, levels = c("5", "4", "3", "2", "1", "N/A"))

# Define the color palette
color_palette <- c("N/A" = "azure4", "1" = "#ff6666", "2" = "#ff9999",
                   "3" = "lightgrey", "4" = "#66ff66", "5" = "#33cc33")

# Define custom labels for the legend
custom_labels <- c("N/A" = "N/A",
                   "1" = "Strongly disagree",
                   "2" = "Disagree",
                   "3" = "No opinion",
                   "4" = "Agree",
                   "5" = "Strongly agree")

# Create the stacked bar plot with custom colors and legend labels
diagnosis_graph <- ggplot(d_long, aes(x = Diagnosis, fill = Rating)) +
  geom_bar(position = position_stack(reverse = TRUE)) +
  scale_fill_manual(values = color_palette, labels = custom_labels) +
  labs(
    title = "Willingness to engage with the pilot VR system as a ... for the purpose of mental health di
    x = NULL, # Remove x-axis title
    y = "Count", # Y-axis title
    fill = "Rating"
  ) +
  theme_minimal() +
  coord_flip() +  # Ensure bars are horizontal
  theme(
    plot.title = element_text(size = 10, face = "bold", hjust = 0.5), # Title size and alignment
    axis.title.x = element_text(size = 10),  # Axis title size
    axis.title.y = element_text(size = 10),  # Y-axis title size
    axis.text.x = element_text(size = 10),   # Axis text size
    axis.text.y = element_text(size = 10),   # Axis text size
    legend.title = element_text(size = 10),  # Legend title size
    legend.text = element_text(size = 10),   # Legend text size
    legend.key.size = unit(0.3, "lines")     # Smaller legend keys
```

```
  )

print(diagnosis_graph)
```

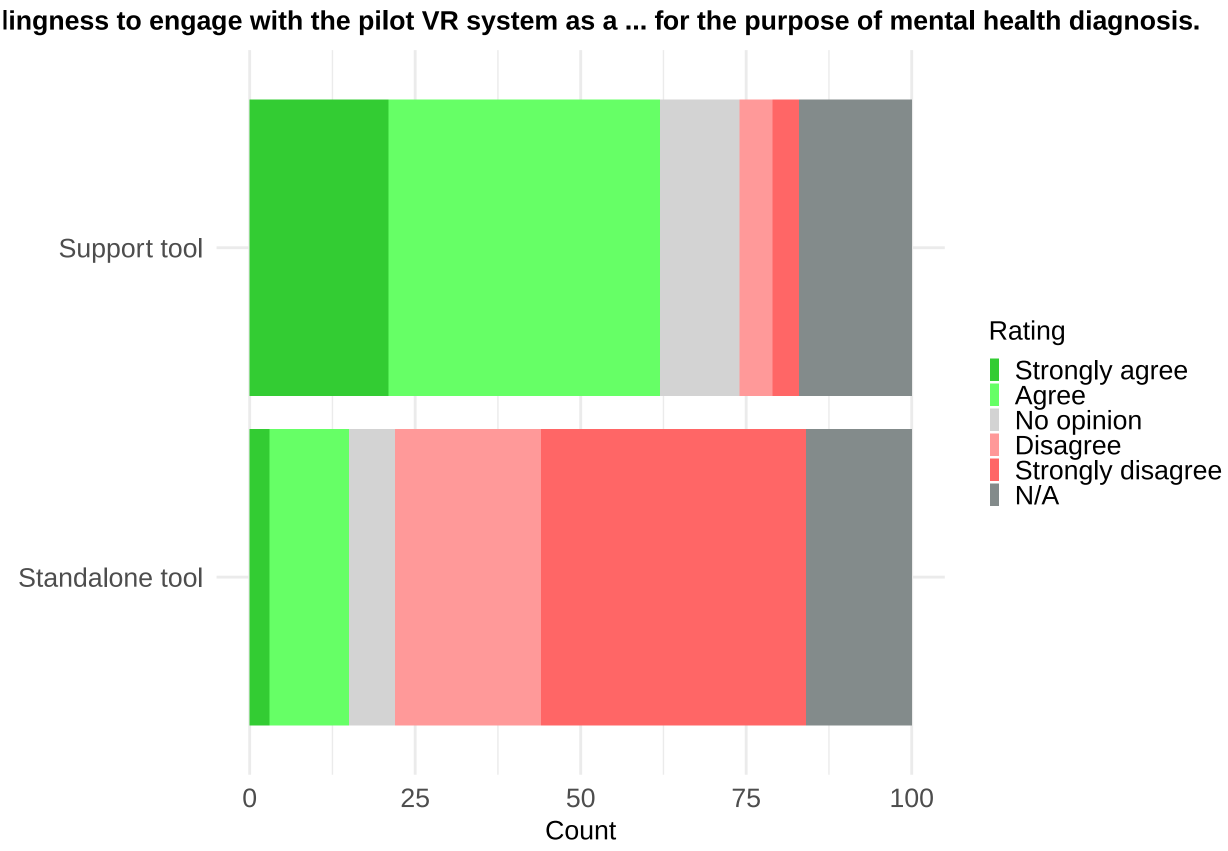


```
# Save in high resolution
png("Diagnosis_Figure.png", units="px", width=2400, height=620, res=300)
print (diagnosis_graph)
dev.off()
```

```
## pdf
##   2
```

Analyses

```
### Wilcoxon signed rank test on standalone tool vs. support tool acceptance.
# Remove rows with missing values in either variable
cleaned_data <- na.omit(d[, c("Willing_diagnosis_HP", "Willing_diagnosis_NHP")])

## Perform the Wilcoxon signed-rank test
tool_comp <- wilcox.test(cleaned_data$Willing_diagnosis_HP,
                          cleaned_data$Willing_diagnosis_NHP,
                          paired = TRUE)
```

```r
# Print the result
print(tool_comp)
```

```
##
##  Wilcoxon signed rank test with continuity correction
##
## data:  cleaned_data$Willing_diagnosis_HP and cleaned_data$Willing_diagnosis_NHP
## V = 1770, p-value = 1.366e-11
## alternative hypothesis: true location shift is not equal to 0
```

```r
## Reshape data to long format to calculate effect size
cleaned_data_long <- cleaned_data %>%
  pivot_longer(
    cols = c(Willing_diagnosis_HP, Willing_diagnosis_NHP),
    names_to = "Condition",
    values_to = "Willingness"
  )

# Calculate effect size
effect_size_w <- cleaned_data_long %>%
  wilcox_effsize(Willingness ~ Condition, paired = TRUE, ci = TRUE)


# Print the result
print(effect_size_w)
```

```
## # A tibble: 1 x 9
##   .y.         group1     group2 effsize    n1    n2 conf.low conf.high magnitude
## * <chr>       <chr>      <chr>    <dbl> <int> <int>    <dbl>     <dbl> <ord>
## 1 Willingness Willing_d~ Willi~   0.808    83    83     0.76      0.84 large
```

```r
median(cleaned_data$Willing_diagnosis_HP, na.rm = T)
```

```
## [1] 4
```

```r
quantile(cleaned_data$Willing_diagnosis_HP, na.rm = T)
```

```
##   0%  25%  50%  75% 100%
##  1.0  3.5  4.0  4.5  5.0
```

```r
median(cleaned_data$Willing_diagnosis_NHP, na.rm = T)
```

```
## [1] 2
```

```r
quantile(cleaned_data$Willing_diagnosis_NHP, na.rm = T)
```

```
##   0%  25%  50%  75% 100%
##    1    1    2    3    5
```

Acceptability of the system - Dimensions of the Theoretical Framework of Acceptability (TFA)

Descriptives

General acceptability

```
# General acceptability
mean(d$Acceptable, na.rm = T)
```

```
## [1] 4.369048
```

```
sd(d$Acceptable, na.rm = T)
```

```
## [1] 0.6358708
```

```
sum(d$Acceptable >3 , na.rm = T)     # Number of those finding the system unacceptable (defined as scor
```

```
## [1] 81
```

```
sum(d$Acceptable!="", na.rm = T)     # Total number of values
```

```
## [1] 84
```

```
sum(d$Acceptable >3 , na.rm = T) / sum(d$Acceptable!="", na.rm = T) * 100 # Percentage
```

```
## [1] 96.42857
```

Affective attitude - Like or dislike

```
mean(d$Like_dislike, na.rm = T)
```

```
## [1] 4.180723
```

```
sd(d$Like_dislike, na.rm = T)
```

```
## [1] 0.8136725
```

```
sum(d$Like_dislike >3 , na.rm = T)     # Number of those finding the system unacceptable
```

```
## [1] 74
```

```
sum(d$Like_dislike!="", na.rm = T)     # Total number of values
```

```
## [1] 83
```

```r
sum(d$Like_dislike >3 , na.rm = T) / sum(d$Like_dislike!="", na.rm = T) * 100
```

```
## [1] 89.15663
```

Affective attitude - Comfort

```r
mean(d$Comfortable, na.rm = T)
```

```
## [1] 3.571429
```

```r
sd(d$Comfortable, na.rm = T)
```

```
## [1] 0.9729562
```

```r
sum(d$Comfortable >3 , na.rm = T)      # Number of those finding the system unacceptable
```

```
## [1] 57
```

```r
sum(d$Comfortable!="", na.rm = T)      # Total number of values
```

```
## [1] 84
```

```r
sum(d$Comfortable >3 , na.rm = T) / sum(d$Comfortable!="", na.rm = T) * 100
```

```
## [1] 67.85714
```

Burden

```r
mean(d$Effort_rev, na.rm = T)
```

```
## [1] 3.809524
```

```r
sd(d$Effort_rev, na.rm = T)
```

```
## [1] 0.842775
```

```r
sum(d$Effort_rev >3 , na.rm = T)      # Number of those finding the system unacceptable
```

```
## [1] 71
```

```r
sum(d$Effort_rev!="", na.rm = T)      # Total number of values
```

```
## [1] 84
```

```r
sum(d$Effort_rev >3 , na.rm = T) / sum(d$Effort!="", na.rm = T) * 100
```

```
## [1] 84.52381
```

Ethicality

```r
mean(d$Moral_ethical_rev, na.rm = T)
```

```
## [1] 3.619048
```

```r
sd(d$Moral_ethical_rev, na.rm = T)
```

```
## [1] 1.085554
```

```r
sum(d$Moral_ethical_rev >3 , na.rm = T)      # Number of those finding the system unacceptable
```

```
## [1] 53
```

```r
sum(d$Moral_ethical_rev!="", na.rm = T)      # Total number of values
```

```
## [1] 84
```

```r
sum(d$Moral_ethical_rev >3 , na.rm = T) / sum(d$Moral_ethical_rev!="", na.rm = T) * 100
```

```
## [1] 63.09524
```

Intervention coherence

```r
mean(d$Clarity_diagnosis, na.rm = T)
```

```
## [1] 3.702381
```

```r
sd(d$Clarity_diagnosis, na.rm = T)
```

```
## [1] 0.9285273
```

```r
sum(d$Clarity_diagnosis >3 , na.rm = T)      # Number of those finding the system unacceptable
```

```
## [1] 60
```

```r
sum(d$Clarity_diagnosis!="", na.rm = T)      # Total number of values
```

```
## [1] 84
```

```r
sum(d$Clarity_diagnosis >3 , na.rm = T) / sum(d$Clarity_diagnosis!="", na.rm = T) * 100
```

```
## [1] 71.42857
```

Opportunity cost

```r
mean(d$Opportunity_cost_rev, na.rm = T)
```

```
## [1] 3.202381
```

```r
sd(d$Opportunity_cost_rev, na.rm = T)
```

```
## [1] 1.14891
```

```r
sum(d$Opportunity_cost_rev >3 , na.rm = T)      # Number of those finding the system unacceptable
```

```
## [1] 43
```

```r
sum(d$Opportunity_cost_rev!="", na.rm = T)      # Total number of values
```

```
## [1] 84
```

```r
sum(d$Opportunity_cost_rev >3 , na.rm = T) / sum(d$Opportunity_cost_rev!="", na.rm = T) * 100
```

```
## [1] 51.19048
```

Perceived effectiveness

```r
mean(d$Improved_mental_state, na.rm = T)
```

```
## [1] 2.869048
```

```r
sd(d$Improved_mental_state, na.rm = T)
```

```
## [1] 0.8327594
```

```r
sum(d$Improved_mental_state >3 , na.rm = T)      # Number of those finding the system unacceptable
```

```
## [1] 19
```

```r
sum(d$Improved_mental_state!="", na.rm = T)      # Total number of values
```

```
## [1] 84
```

```r
sum(d$Improved_mental_state >3 , na.rm = T) / sum(d$Improved_mental_state!="", na.rm = T) * 100
```

```
## [1] 22.61905
```

Self-efficacy - Taken from the SUS questionnaire (item 9)

```r
mean(d$Confident_SUS9, na.rm = T)
```

```
## [1] 2.79798
```

```r
sd(d$Confident_SUS9, na.rm = T)
```

```
## [1] 0.9997938
```

```r
sum(d$Confident_SUS9 >3 , na.rm = T)     # Number of those finding the system unacceptable
```

```
## [1] 21
```

```r
sum(d$Confident_SUS9!="", na.rm = T)     # Total number of values
```

```
## [1] 99
```

```r
sum(d$Confident_SUS9 >3 , na.rm = T) / sum(d$Confident_SUS9!="", na.rm = T) * 100
```

```
## [1] 21.21212
```

Visualization

Figure 7. Self-reported acceptability of the pilot VR system assessed using constructs from the Theoretical Framework of Acceptability.

```r
# Reshape data from wide to long format
d_long <- d %>%
  pivot_longer(cols = c(Acceptable, Like_dislike, Comfortable, Improved_mental_state,
                        Clarity_diagnosis, Confident_SUS9, Effort_rev,
                        Moral_ethical_rev, Opportunity_cost_rev),  # New variables
               names_to = "Variable",  # Rename to match new variables
               values_to = "Rating") %>%
  # Rename the variables if you want custom labels
  mutate(Variable = recode(Variable,
                          "Acceptable" = "General acceptability",
                          "Like_dislike" = "Like or dislike",
                          "Comfortable" = "Comfort",
                          "Improved_mental_state" = "Perceived effectiveness",
                          "Clarity_diagnosis" = "Intervention coherence",
                          "Confident_SUS9" = "Self-efficacy",
                          "Effort_rev" = "Burden",
                          "Moral_ethical_rev" = "Ethicality",
```

```r
                              "Opportunity_cost_rev" = "Opportunity cost"),
         Rating = ifelse(is.na(Rating), "N/A", Rating))  # Replace NA with "N/A"))

# Convert Rating to numeric for mean calculation
d_long <- d_long %>%
  mutate(Rating_num = as.numeric(factor(Rating, levels = c("1", "2", "3", "4", "5", "N/A"))))

# Calculate mean ratings for each variable
mean_ratings <- d_long %>%
  group_by(Variable) %>%
  summarise(mean_rating = mean(Rating_num, na.rm = TRUE)) %>%
  arrange(mean_rating)

# Reorder the Variable factor levels based on mean ratings
d_long <- d_long %>%
  mutate(Variable = factor(Variable, levels = mean_ratings$Variable))

# Convert Rating to a factor with reversed levels for stacking
d_long$Rating <- factor(d_long$Rating, levels = c("5", "4", "3", "2", "1", "N/A"))

# Define the color palette
color_palette <- c("N/A" = "azure4", "1" = "#ff6666", "2" = "#ff9999",
                   "3" = "lightgrey", "4" = "#66ff66", "5" = "#33cc33")

# Define custom labels for the legend
custom_labels <- c("N/A" = "N/A",
                   "1" = "1",
                   "2" = "2",
                   "3" = "No opinion",
                   "4" = "4",
                   "5" = "5")

# Create the stacked bar plot with custom colors and legend labels
TFA_graph <- ggplot(d_long, aes(x = Variable, fill = Rating)) +
  geom_bar(position = position_stack(reverse = TRUE)) +
  scale_fill_manual(values = color_palette, labels = custom_labels) +
  labs(
    title = "Constructs of acceptability",  # Update graph title
    x = NULL,  # Remove x-axis title
    y = "Count",  # Y-axis title
  ) +
  theme_minimal() +
  coord_flip() +  # Ensure bars are horizontal
  theme(
    plot.title = element_text(size = 10, face = "bold", hjust = 0.5),  # Title size and alignment
    axis.title.x = element_text(size = 10),  # Axis title size
    axis.title.y = element_text(size = 10),  # Y-axis title size
    axis.text.x = element_text(size = 10),   # Axis text size
    axis.text.y = element_text(size = 10),   # Axis text size
    legend.title = element_text(size = 10),  # Legend title size
    legend.text = element_text(size = 10)    # Legend text size
  )
```

```r
print(TFA_graph)
```

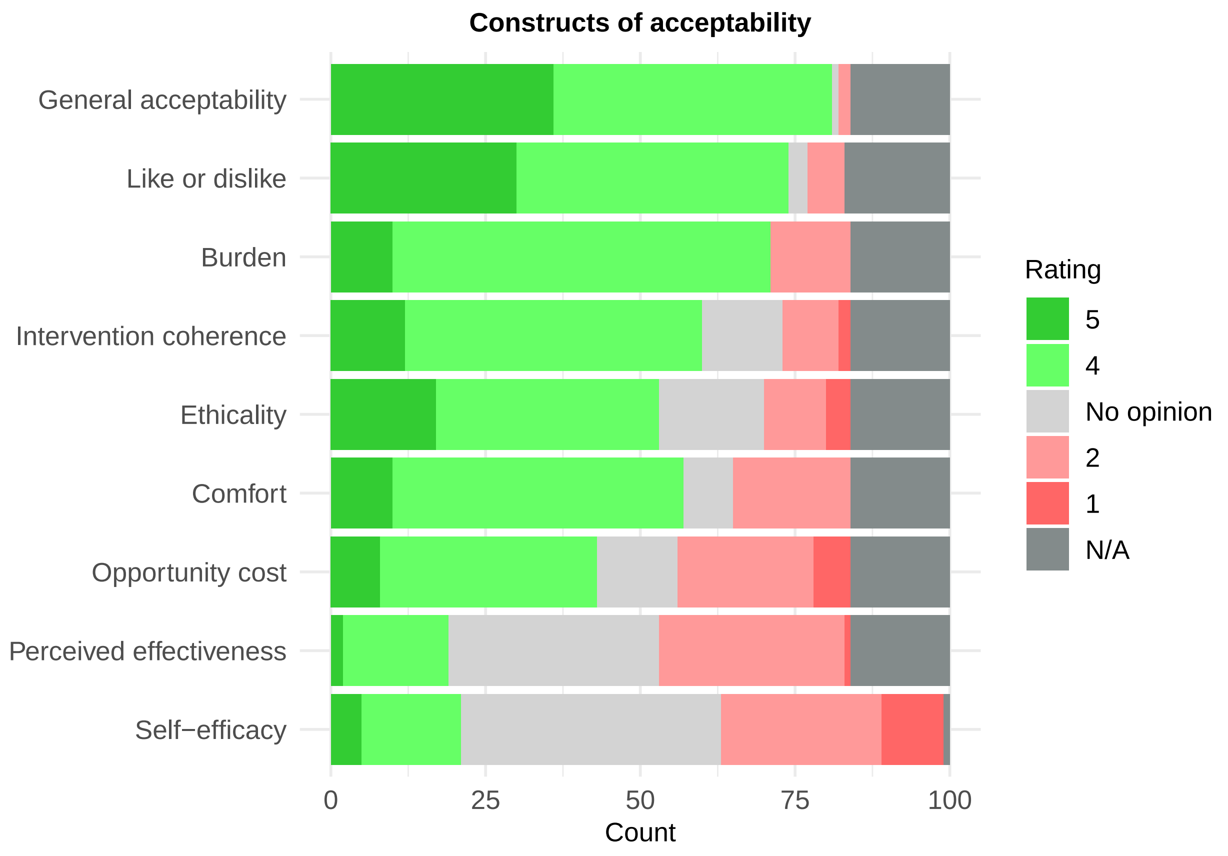


```r
# Save in high resolution
png("TFA_graph.png", units="px", width=2600, height=1000, res=300)
print(TFA_graph)
dev.off()
```

```
## pdf
##   2
```

Analyses

Ordinal regression model on TFA dimensions

```r
## General acceptability
# Fitting ordinal regression model
Gen_accep_demographics_ordinal <- clm(as.factor(d$Acceptable) ~
                                        d$Age + d$Gender + d$Education, na.rm = T)

# Summary of the ordinal regression model
summary(Gen_accep_demographics_ordinal)
```

```
## formula: as.factor(d$Acceptable) ~ d$Age + d$Gender + d$Education
##
##  link  threshold nobs logLik AIC    niter max.grad cond.H
##  logit flexible  84   -66.04 144.09 7(1)  9.93e-08 7.3e+05
##
## Coefficients:
##             Estimate Std. Error z value Pr(>|z|)
## d$Age         0.1751     0.1905   0.919   0.3581
## d$Gender     -1.3310     0.5877  -2.265   0.0235 *
## d$Education  -0.5291     0.2732  -1.937   0.0527 .
## ---
## Signif. codes:  0 '***' 0.001 '**' 0.01 '*' 0.05 '.' 0.1 ' ' 1
##
## Threshold coefficients:
##     Estimate Std. Error z value
## 2|3  -10.837      3.979  -2.723
## 3|4  -10.418      3.958  -2.632
## 4|5   -6.654      3.866  -1.721
## (16 observations deleted due to missingness)
```

```r
confint(Gen_accep_demographics_ordinal)
```

```
##                  2.5 %      97.5 %
## d$Age       -0.1944341  0.56248199
## d$Gender    -2.5428287 -0.21016934
## d$Education -1.0970170 -0.01231931
```

```r
mean(d$Acceptable[d$Gender=="1"], na.rm = T)
```

```
## [1] 4.647059
```

```r
sd(d$Acceptable[d$Gender=="1"], na.rm = T)
```

```
## [1] 0.4925922
```

```r
mean(d$Acceptable[d$Gender=="2"], na.rm = T)
```

```
## [1] 4.298507
```

```r
sd(d$Acceptable[d$Gender=="2"], na.rm = T)
```

```
## [1] 0.6516861
```

```r
# Testing the assumptions of proportional odds
Gen_accep_demographics_ordinal_PO <- polr(as.factor(Acceptable) ~
                                            Age + Gender + Education,
               data = d, Hess = T)

# Brant test
brant(Gen_accep_demographics_ordinal_PO)
```

```
## ---------------------------------------------
## Test for X2  df  probability
## ---------------------------------------------
## Omnibus      6.16    6   0.41
## Age      4.29    2   0.12
## Gender       0   2   1
## Education    0.92    2   0.63
## ---------------------------------------------
##
## H0: Parallel Regression Assumption holds
```

```
## Affective attitude
# Like-dislike
TFA_affective_demographics <- clm(as.factor(d$Like_dislike) ~ d$Age + d$Gender + d$Education, na.rm = T
summary(TFA_affective_demographics)
```

```
## formula: as.factor(d$Like_dislike) ~ d$Age + d$Gender + d$Education
##
##  link  threshold nobs logLik AIC    niter max.grad cond.H
##  logit flexible  83   -82.38 176.77 6(1)  8.76e-08 7.0e+05
##
## Coefficients:
##             Estimate Std. Error z value Pr(>|z|)
## d$Age        -0.2977     0.1824  -1.632    0.103
## d$Gender     -0.4609     0.5332  -0.865    0.387
## d$Education   0.1640     0.2550   0.643    0.520
##
## Threshold coefficients:
##     Estimate Std. Error z value
## 2|3   -7.686      3.649  -2.107
## 3|4   -7.236      3.638  -1.989
## 4|5   -4.454      3.568  -1.248
## (17 observations deleted due to missingness)
```

```
confint(TFA_affective_demographics)
```

```
##                  2.5 %     97.5 %
## d$Age       -0.6654785 0.05283245
## d$Gender    -1.5245520 0.58025480
## d$Education -0.3371345 0.67126358
```

```
table(d$Like_dislike)
```

```
##
##  2  3  4  5
##  6  3 44 30
```

```
mean(d$Like_dislike[d$Group == "Control"], na.rm = TRUE)
```

```
## [1] 4.375
```

```r
sd(d$Like_dislike[d$Group == "Control"], na.rm = TRUE)
```

```
## [1] 0.5856182
```

```r
mean(d$Like_dislike[d$Group == "Depressed"], na.rm = TRUE)
```

```
## [1] 4
```

```r
sd(d$Like_dislike[d$Group == "Depressed"], na.rm = TRUE)
```

```
## [1] 0.9511897
```

```r
# Comfort
TFA_comfort_demographics <- clm(as.factor(d$Comfortable) ~ d$Age + d$Gender + d$Education, na.rm = T)
summary(TFA_comfort_demographics)
```

```
## formula: as.factor(d$Comfortable) ~ d$Age + d$Gender + d$Education
##
##  link  threshold nobs logLik AIC    niter max.grad cond.H
##  logit flexible  84   -94.57 201.14 7(0)  4.53e-08 6.1e+05
##
## Coefficients:
##             Estimate Std. Error z value Pr(>|z|)
## d$Age       -0.04499    0.16858  -0.267    0.790
## d$Gender    -0.71782    0.56059  -1.280    0.200
## d$Education  0.11597    0.23823   0.487    0.626
##
## Threshold coefficients:
##     Estimate Std. Error z value
## 2|3   -1.711      3.353  -0.510
## 3|4   -1.222      3.350  -0.365
## 4|5    1.594      3.351   0.476
## (16 observations deleted due to missingness)
```

```r
confint(TFA_comfort_demographics)
```

```
##                  2.5 %    97.5 %
## d$Age       -0.3776510 0.2865678
## d$Gender    -1.8417172 0.3670108
## d$Education -0.3601082 0.5824468
```

```r
table(d$Comfortable)
```

```
##
##  2  3  4  5
## 19  8 47 10
```

```r
mean(d$Comfortable[d$Group == "Control"], na.rm = TRUE)
```

```
## [1] 3.825
```

```r
sd(d$Comfortable[d$Group == "Control"], na.rm = TRUE)
```

```
## [1] 0.7807787
```

```r
mean(d$Comfortable[d$Group == "Depressed"], na.rm = TRUE)
```

```
## [1] 3.340909
```

```r
sd(d$Comfortable[d$Group == "Depressed"], na.rm = TRUE)
```

```
## [1] 1.077102
```

```r
## Burden
TFA_burden_demographics <- clm(as.factor(d$Effort_rev) ~ d$Age + d$Gender + d$Education, na.rm = T)
summary(TFA_burden_demographics)
```

```
## formula: as.factor(d$Effort_rev) ~ d$Age + d$Gender + d$Education
##
##  link  threshold nobs logLik AIC    niter max.grad cond.H
##  logit flexible  84   -60.35 130.70 6(0)  1.13e-07 5.0e+05
##
## Coefficients:
##             Estimate Std. Error z value Pr(>|z|)
## d$Age         0.1365     0.2026   0.674  0.50059
## d$Gender     -1.8655     0.6737  -2.769  0.00562 **
## d$Education  -0.1857     0.2923  -0.635  0.52529
## ---
## Signif. codes:  0 '***' 0.001 '**' 0.01 '*' 0.05 '.' 0.1 ' ' 1
##
## Threshold coefficients:
##     Estimate Std. Error z value
## 2|4  -5.0794     4.3185  -1.176
## 4|5  -0.9453     4.2488  -0.222
## (16 observations deleted due to missingness)
```

```r
confint(TFA_burden_demographics)
```

```
##                  2.5 %     97.5 %
## d$Age       -0.2599804  0.5447234
## d$Gender    -3.2571089 -0.5862032
## d$Education -0.7702330  0.3786999
```

```r
table(d$Effort_rev)
```

```
##
##  2  4  5
## 13 61 10
```

```r
mean(d$Effort_rev[d$Gender == 1], na.rm = TRUE)
```

```
## [1] 4.294118
```

```r
sd(d$Effort_rev[d$Gender == 1], na.rm = TRUE)
```

```
## [1] 0.4696682
```

```r
mean(d$Effort_rev[d$Gender == 2], na.rm = TRUE)
```

```
## [1] 3.686567
```

```r
sd(d$Effort_rev[d$Gender == 2], na.rm = TRUE)
```

```
## [1] 0.8740182
```

```r
## Ethicality
TFA_ethical_demographics <- clm(as.factor(d$Moral_ethical_rev) ~ d$Age + d$Gender + d$Education, na.rm =
summary(TFA_ethical_demographics)
```

```
## formula: as.factor(d$Moral_ethical_rev) ~ d$Age + d$Gender + d$Education
##
##  link  threshold nobs logLik  AIC    niter max.grad cond.H
##  logit flexible  84   -116.02 246.05 5(0)  3.80e-09 1.1e+06
##
## Coefficients:
##              Estimate Std. Error z value Pr(>|z|)
## d$Age          0.3200     0.1655   1.934   0.0532 .
## d$Gender       0.2409     0.5006   0.481   0.6304
## d$Education  -0.1663     0.2524  -0.659   0.5101
## ---
## Signif. codes:  0 '***' 0.001 '**' 0.01 '*' 0.05 '.' 0.1 ' ' 1
##
## Threshold coefficients:
##     Estimate Std. Error z value
## 1|2    2.063      3.603   0.573
## 2|3    3.459      3.584   0.965
## 3|4    4.562      3.597   1.268
## 4|5    6.558      3.641   1.801
## (16 observations deleted due to missingness)
```

```r
confint(TFA_ethical_demographics)
```

```
##                      2.5 %    97.5 %
## d$Age       -0.0008598237 0.6537660
## d$Gender    -0.7429555099 1.2292814
## d$Education -0.6641791010 0.3275519
```

```
table(d$Moral_ethical_rev)
```

```
##
##  1  2  3  4  5
##  4 10 17 36 17
```

```
## Intervention coherence
TFA_clarity_demographics <- clm(as.factor(d$Clarity_diagnosis) ~ d$Age + d$Gender + d$Education, na.rm =
summary(TFA_clarity_demographics)
```

```
## formula: as.factor(d$Clarity_diagnosis) ~ d$Age + d$Gender + d$Education
##
##  link  threshold nobs logLik  AIC    niter max.grad cond.H
##  logit flexible  84   -101.60 217.20 6(0)  5.51e-12 1.0e+06
##
## Coefficients:
##             Estimate Std. Error z value Pr(>|z|)
## d$Age        0.08541    0.17088   0.500    0.617
## d$Gender    -0.31529    0.55577  -0.567    0.571
## d$Education -0.19348    0.25136  -0.770    0.441
##
## Threshold coefficients:
##     Estimate Std. Error z value
## 1|2 -5.47286    3.77287  -1.451
## 2|3 -3.64814    3.71476  -0.982
## 3|4 -2.66885    3.70478  -0.720
## 4|5  0.06089    3.69131   0.016
## (16 observations deleted due to missingness)
```

```
confint(TFA_clarity_demographics)
```

```
##                  2.5 %    97.5 %
## d$Age       -0.2501625 0.4223496
## d$Gender    -1.4203449 0.7676860
## d$Education -0.6947705 0.2933319
```

```
table(d$Clarity_diagnosis)
```

```
##
##  1  2  3  4  5
##  2  9 13 48 12
```

```
# Opportunity cost
TFA_opportunity_demographics <- clm(as.factor(d$Opportunity_cost_rev) ~ d$Age + d$Gender + d$Education,
summary(TFA_opportunity_demographics)
```

```
## formula: as.factor(d$Opportunity_cost_rev) ~ d$Age + d$Gender + d$Education
##
##  link  threshold nobs logLik  AIC    niter max.grad cond.H
##  logit flexible  84   -118.48 250.96 6(0)  2.77e-13 9.4e+05
```

```
##
## Coefficients:
##             Estimate Std. Error z value Pr(>|z|)
## d$Age       -0.07778    0.15999  -0.486    0.627
## d$Gender    -0.49645    0.51471  -0.965    0.335
## d$Education  0.02329    0.23473   0.099    0.921
##
## Threshold coefficients:
##     Estimate Std. Error z value
## 1|2 -4.89799    3.42394  -1.431
## 2|3 -3.00789    3.38856  -0.888
## 3|4 -2.35105    3.37956  -0.696
## 4|5 -0.03738    3.38115  -0.011
## (16 observations deleted due to missingness)
```

```r
confint(TFA_opportunity_demographics)
```

```
##                  2.5 %    97.5 %
## d$Age       -0.3949394 0.2374382
## d$Gender    -1.5259126 0.5031758
## d$Education -0.4371641 0.4890353
```

```r
table(d$Opportunity_cost_rev)
```

```
##
##  1  2  3  4  5
##  6 22 13 35  8
```

```r
## Perceived effectiveness
TFA_effectiveness_demographics <- clm(as.factor(d$Improved_mental_state) ~ d$Age + d$Gender + d$Educati
summary(TFA_effectiveness_demographics)
```

```
## formula: as.factor(d$Improved_mental_state) ~ d$Age + d$Gender + d$Education
##
##  link  threshold nobs logLik AIC    niter max.grad cond.H
##  logit flexible  84   -99.38 212.76 7(0)  1.86e-07 1.1e+06
##
## Coefficients:
##             Estimate Std. Error z value Pr(>|z|)
## d$Age       -0.11588    0.16249  -0.713    0.476
## d$Gender    -0.75714    0.51772  -1.462    0.144
## d$Education -0.04527    0.24336  -0.186    0.852
##
## Threshold coefficients:
##     Estimate Std. Error z value
## 1|2   -9.247      3.818  -2.422
## 2|3   -5.333      3.668  -1.454
## 3|4   -3.521      3.642  -0.967
## 4|5   -1.006      3.676  -0.274
## (16 observations deleted due to missingness)
```

```r
confint(TFA_effectiveness_demographics)
```

```
##                  2.5 %    97.5 %
## d$Age       -0.442515 0.1997137
## d$Gender    -1.785798 0.2548370
## d$Education -0.530228 0.4302125
```

```r
table(d$Improved_mental_state)
```

```
##
##  1  2  3  4  5
##  1 30 34 17  2
```

```r
## Self-efficacy - from SUS
TFA_efficacy_demographics <- clm(as.factor(d$Confident_SUS9) ~ d$Age + d$Gender + d$Education, na.rm = 
summary(TFA_efficacy_demographics)
```

```
## formula: as.factor(d$Confident_SUS9) ~ d$Age + d$Gender + d$Education
##
##  link  threshold nobs logLik  AIC    niter max.grad cond.H
##  logit flexible  99   -135.56 285.11 5(0)  3.00e-07 1.1e+06
##
## Coefficients:
##             Estimate Std. Error z value Pr(>|z|)
## d$Age        0.06309    0.15310   0.412   0.6803
## d$Gender    -0.87221    0.49288  -1.770   0.0768 .
## d$Education  0.03161    0.22372   0.141   0.8876
## ---
## Signif. codes:  0 '***' 0.001 '**' 0.01 '*' 0.05 '.' 0.1 ' ' 1
##
## Threshold coefficients:
##     Estimate Std. Error z value
## 1|2  -1.8202     3.3386  -0.545
## 2|3  -0.1789     3.3321  -0.054
## 3|4   1.7359     3.3305   0.521
## 4|5   3.4128     3.3532   1.018
## (1 observation deleted due to missingness)
```

```r
confint(TFA_efficacy_demographics)
```

```
##                   2.5 %     97.5 %
## d$Age       -0.2385623 0.36510683
## d$Gender    -1.8579985 0.08212483
## d$Education -0.4063961 0.47537985
```

```r
table(d$Confident_SUS9)
```

```
##
##  1  2  3  4  5
## 10 26 42 16  5
```

Usability

Descriptives

```
mean(d$SUS_Total, na.rm = T)
```

```
## [1] 69.01515
```

```
median(d$SUS_Total, na.rm = T)
```

```
## [1] 70
```

```
sd(d$SUS_Total, na.rm = T)
```

```
## [1] 12.8612
```

```
min(d$SUS_Total, na.rm = T)
```

```
## [1] 37.5
```

```
max(d$SUS_Total, na.rm = T)
```

```
## [1] 97.5
```

```
sum(d$SUS_Total < 50.9, na.rm = T) # number of those rating it below OK level (Bangor et al., 2009)
```

```
## [1] 12
```

```
sum(d$SUS_Total < 68, na.rm = T)   # number of those rating it below average level
```

```
## [1] 41
```

```
sum(d$SUS_Total!="", na.rm = T)    # Total number of values
```

```
## [1] 99
```

```
sum(d$SUS_Total < 68, na.rm = T) / sum(d$SUS_Total!="", na.rm = T) * 100
```

```
## [1] 41.41414
```

Visualization

Figure 8. Density plot of overall usability ratings on the System Usability Scale from all participants.

```r
# Display the plot in R
par(mfrow=c(1,1))
vioplot(d$SUS_Total, horizontal=TRUE, xaxt = "n", col="#33cc33",
        xlab="System Usability Scale score",
        ylab="Density of participants", ylim = c(0, 100))
abline(v = 48.9, col = "red", lty = 2)
# Add rotated, smaller text with vertical alignment
text(49.9 + 2, 0.5, "satisfactory",
     col = "red", srt = 90, cex = 0.6, adj = c(-3.3, -1.3))
```

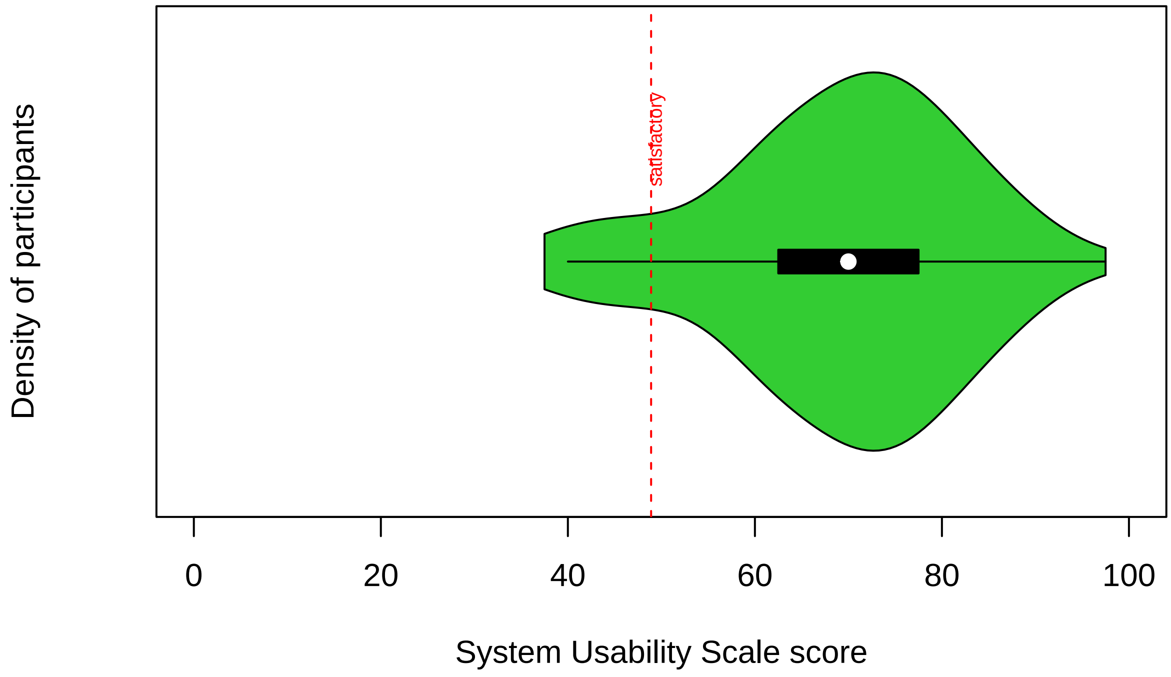


```r
# Save Figure in high resolution
par(mfrow=c(1,1))
png("SUS_Figure_scale.png", units="px", width=2200, height=1600, res=300)
vioplot(d$SUS_Total, horizontal=TRUE, xaxt = "n", col="#33cc33",
        xlab="System Usability Scale score",
        ylab="Density of participants", ylim = c(0, 100))
abline(v = 50.9, col = "red", lty = 2)
# Add rotated, smaller text with vertical alignment
text(49.9 + 2, 0.5, "satisfactory",
     col = "red", srt = 90, cex = 0.6, adj = c(-4.75, -1.50))
dev.off()
```

```
## pdf
##   2
```

Analyses

```r
# Multiple linear regression model on predicting sus with study groups
SUS_demographics <- lm(d$SUS_Total ~ d$Age + d$Education + d$Gender + d$Group)
summary(SUS_demographics)
```

```
##
## Call:
## lm(formula = d$SUS_Total ~ d$Age + d$Education + d$Gender + d$Group)
##
## Residuals:
##     Min      1Q  Median      3Q     Max
## -31.760  -6.518   1.523   8.479  25.727
##
## Coefficients:
##                   Estimate Std. Error t value Pr(>|t|)
## (Intercept)       105.7306    23.0161   4.594 1.35e-05 ***
## d$Age               0.4324     1.0782   0.401   0.6893
## d$Education        -2.1501     1.6068  -1.338   0.1841
## d$Gender           -5.7903     3.3211  -1.744   0.0845 .
## d$GroupDepressed   -2.4241     2.6444  -0.917   0.3617
## ---
## Signif. codes:  0 '***' 0.001 '**' 0.01 '*' 0.05 '.' 0.1 ' ' 1
##
## Residual standard error: 12.8 on 94 degrees of freedom
##   (1 observation deleted due to missingness)
## Multiple R-squared:  0.0496, Adjusted R-squared:  0.009153
## F-statistic: 1.226 on 4 and 94 DF,  p-value: 0.305
```

```r
confint(SUS_demographics)
```

```
##                        2.5 %      97.5 %
## (Intercept)        60.031674 151.4295476
## d$Age              -1.708425   2.5731255
## d$Education        -5.340476   1.0402119
## d$Gender          -12.384305   0.8037682
## d$GroupDepressed   -7.674634   2.8265128
```

```r
# Multiple linear regression model on predicting SUS with SSQ
SUS_pred <- lm(SUS_Total ~ Age + Education + Gender + SSQ_Total, data = d)
summary(SUS_pred)
```

```
##
## Call:
## lm(formula = SUS_Total ~ Age + Education + Gender + SSQ_Total,
##     data = d)
##
## Residuals:
##     Min      1Q  Median      3Q     Max
## -34.734  -6.635  -0.075   8.599  23.067
##
```

```
## Coefficients:
##             Estimate Std. Error t value Pr(>|t|)
## (Intercept) 109.91220   21.87325   5.025 2.39e-06 ***
## Age           0.15763    1.04327   0.151  0.88023
## Education    -1.93969    1.52112  -1.275  0.20539
## Gender       -5.05555    3.21337  -1.573  0.11901
## SSQ_Total    -0.12388    0.04518  -2.742  0.00731 **
## ---
## Signif. codes:  0 '***' 0.001 '**' 0.01 '*' 0.05 '.' 0.1 ' ' 1
##
## Residual standard error: 12.37 on 94 degrees of freedom
##   (1 observation deleted due to missingness)
## Multiple R-squared:  0.1121, Adjusted R-squared:  0.07433
## F-statistic: 2.967 on 4 and 94 DF,  p-value: 0.02344
```

```
confint(SUS_pred)
```

```
##                   2.5 %       97.5 %
## (Intercept)  66.4823520 153.34204375
## Age          -1.9138119   2.22906741
## Education    -4.9599096   1.08052672
## Gender      -11.4357710   1.32467707
## SSQ_Total    -0.2135788  -0.03417289
```

Cybersickness

Descriptives

```
## Total scale
# SSQ Total score
mean(d$SSQ_Total, na.rm = T)
```

```
## [1] 30.48667
```

```
median(d$SSQ_Total, na.rm = T)
```

```
## [1] 22.44
```

```
sd(d$SSQ_Total, na.rm = T)
```

```
## [1] 28.03022
```

```
min(d$SSQ_Total, na.rm = T)
```

```
## [1] 0
```

```r
max(d$SSQ_Total, na.rm = T)
```

```
## [1] 119.68
```

```r
sum(d$SSQ_Total >= 20, na.rm = T)
```

```
## [1] 55
```

```r
sum(d$SSQ_Total!="", na.rm = T)     # Total number of values
```

```
## [1] 99
```

```r
sum(d$SSQ_Total >= 20, na.rm = T) / sum(d$SSQ_Total!="", na.rm = T) * 100
```

```
## [1] 55.55556
```

```r
## Subscales
# Nausea
mean(d$Nausea, na.rm = T)
```

```
## [1] 21.58545
```

```r
median(d$Nausea, na.rm = T)
```

```
## [1] 19.08
```

```r
sd(d$Nausea, na.rm = T)
```

```
## [1] 21.9579
```

```r
sum(d$Nausea >= 20, na.rm = T)
```

```
## [1] 35
```

```r
sum(d$Nausea!="", na.rm = T)     # Total number of values
```

```
## [1] 99
```

```r
sum(d$Nausea >= 20, na.rm = T) / sum(d$Nausea!="", na.rm = T) * 100
```

```
## [1] 35.35354
```

```r
# Oculomotor disturbance
mean(d$Oculomotor, na.rm = T)
```

```
## [1] 26.72141
```

```r
median(d$Oculomotor, na.rm = T)
```

```
## [1] 22.74
```

```r
sd(d$Oculomotor, na.rm = T)
```

```
## [1] 24.62979
```

```r
sum(d$Oculomotor >= 20, na.rm = T)
```

```
## [1] 52
```

```r
sum(d$Oculomotor!="", na.rm = T)      # Total number of values
```

```
## [1] 99
```

```r
sum(d$Oculomotor >= 20, na.rm = T) / sum(d$Oculomotor!="", na.rm = T) * 100
```

```
## [1] 52.52525
```

```r
# Disorientation
mean(d$Disorientation, na.rm = T)
```

```
## [1] 32.90182
```

```r
median(d$Disorientation, na.rm = T)
```

```
## [1] 13.92
```

```r
sd(d$Disorientation, na.rm = T)
```

```
## [1] 39.84367
```

```r
sum(d$Disorientation >= 20, na.rm = T)
```

```
## [1] 45
```

```r
sum(d$Disorientation!="", na.rm = T)      # Total number of values
```

```
## [1] 99
```

```r
sum(d$Disorientation >= 20, na.rm = T) / sum(d$Disorientation!="", na.rm = T) * 100
```

```
## [1] 45.45455
```

Visualization

Regression, between group difference

Figure 9. Boxplot of self-reported cybersickness severity scores for the 'Healthy control' (HC) and 'Depressive symptoms' (DS) study groups - measured by the Simulator Sickness Questionnaire (A), and its three subscales of Nausea (B), Oculomotor disturbances (C) and Disorientation (D).

```r
## SSQ Total
boxplot(d$SSQ_Total ~ d$Group,
        xlab = "Group",
        ylab = "SSQ total score",
        col = c("grey", "#33cc33"),
        ylim = c(0, 235),             # Set Y-axis limits to the limits of the scale
        xaxt = 'n')                   # Disable default x-axis)
# Custom x-axis with manually set labels
axis(1,
     at = c(1, 2),  # Positions of the groups on the x-axis
     labels = c("HC", "DS"))  # Custom labels for 'depressed' and 'control'
# Add horizontal line at literature average
abline(h = 28, col = "red", lty = 2) # Literature average based on Saredakis et al., 2020
# Add left-aligned rotated text with custom positioning
text(x = 2.75, y = 32, "literature average",
     col = "red", srt = 90, cex = 0.8, xpd = TRUE, adj = c(0, 0))
```

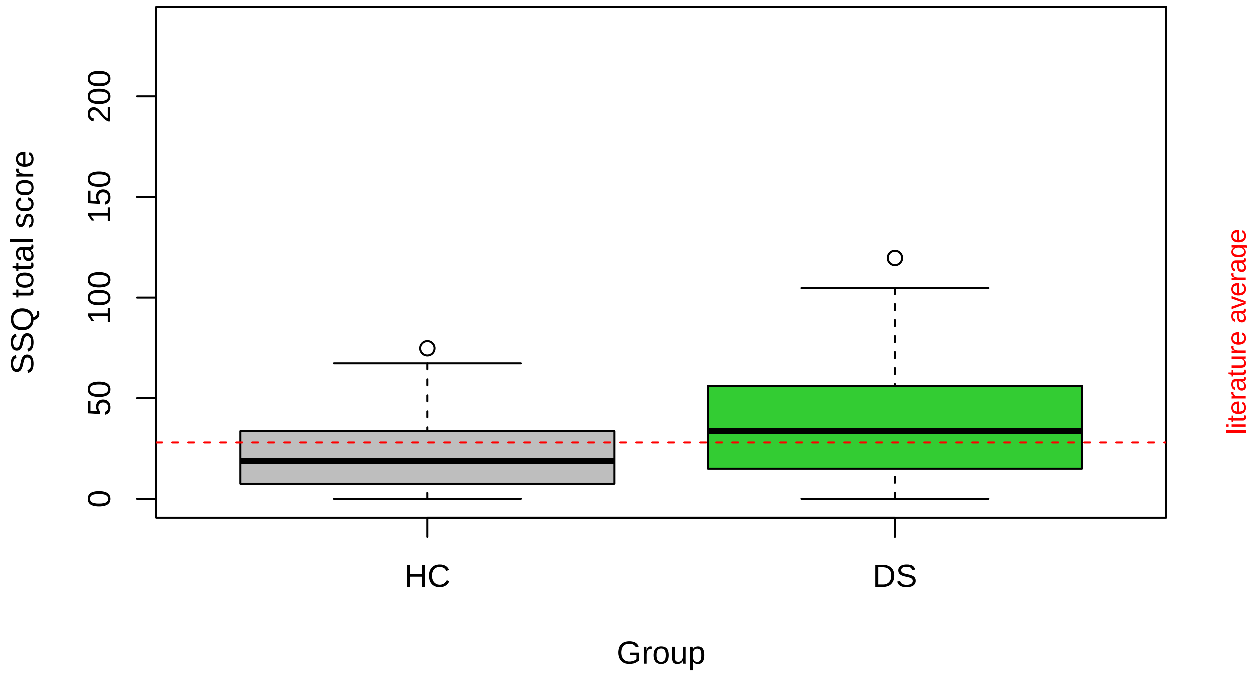

```r
SSQ_boxplotA <- recordPlot()
#print(SSQ_boxplotA)

# Save figure
png("SSQ_Total_figure.png", units="px", width=1000, height=1800, res=300)
print(SSQ_boxplotA)
dev.off()
```

```
## pdf
##   2
```

```r
## Nausea
boxplot(d$Nausea ~ d$Group,
        xlab = "Group",
        ylab = "SSQ nausea score",
        col = c("grey", "#33cc33"),
        ylim = c(0, 200),               # Set Y-axis limits to the limits of the subscale
        xaxt = 'n')                     # Disable default x-axis)
# Custom x-axis with manually set labels
axis(1,
     at = c(1, 2),  # Positions of the groups on the x-axis
     labels = c("HC", "DS"))  # Custom labels for 'depressed' and 'control'
# Add horizontal line at literature average
abline(h = 16.72, col = "red", lty = 2) # Literature average based on Saredakis et al., 2020
# Add left-aligned rotated text with custom positioning
text(x = 2.75, y = 23, "literature average",
     col = "red", srt = 90, cex = 0.8, xpd = TRUE, adj = c(0, 0))
```

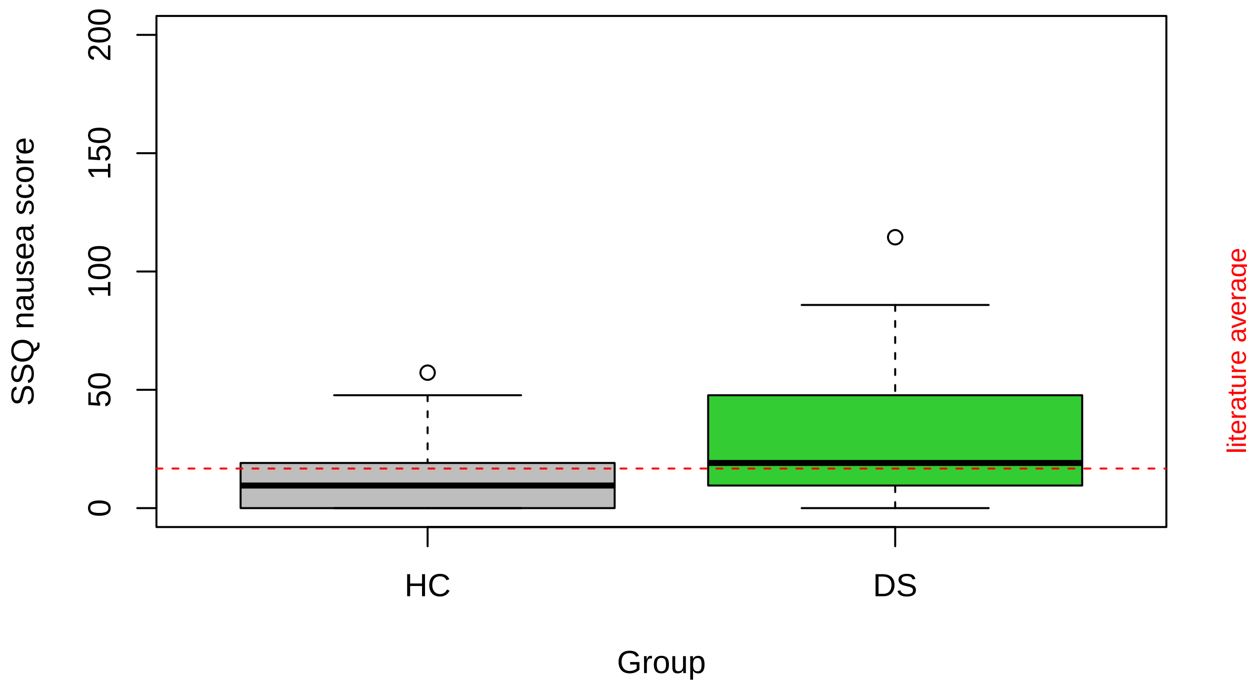


```
SSQ_boxplotB <- recordPlot()
#print(SSQ_boxplotB)

# Save figure
png("SSQ_Nausea_figure.png", units="px", width=1000, height=1800, res=300)
print(SSQ_boxplotB)
dev.off()
```

```
## pdf
##   2
```

```
## Oculomotor
boxplot(d$Oculomotor ~ d$Group,
        xlab = "Group",
        ylab = "SSQ oculomotor disturbance score",
        col = c("grey", "#33cc33"),
        ylim = c(0, 159),             # Set Y-axis limits to the limits of the subscale
        xaxt = 'n')                   # Disable default x-axis)
# Custom x-axis with manually set labels
axis(1,
     at = c(1, 2),  # Positions of the groups on the x-axis
     labels = c("HC", "DS"))  # Custom labels for 'depressed' and 'control'
# Add horizontal line at literature average
abline(h = 17.09, col = "red", lty = 2) # Literature average based on Saredakis et al., 2020
# Add left-aligned rotated text with custom positioning
```

```r
text(x = 2.75, y = 23, "literature average",
     col = "red", srt = 90, cex = 0.8, xpd = TRUE, adj = c(0, 0))
```

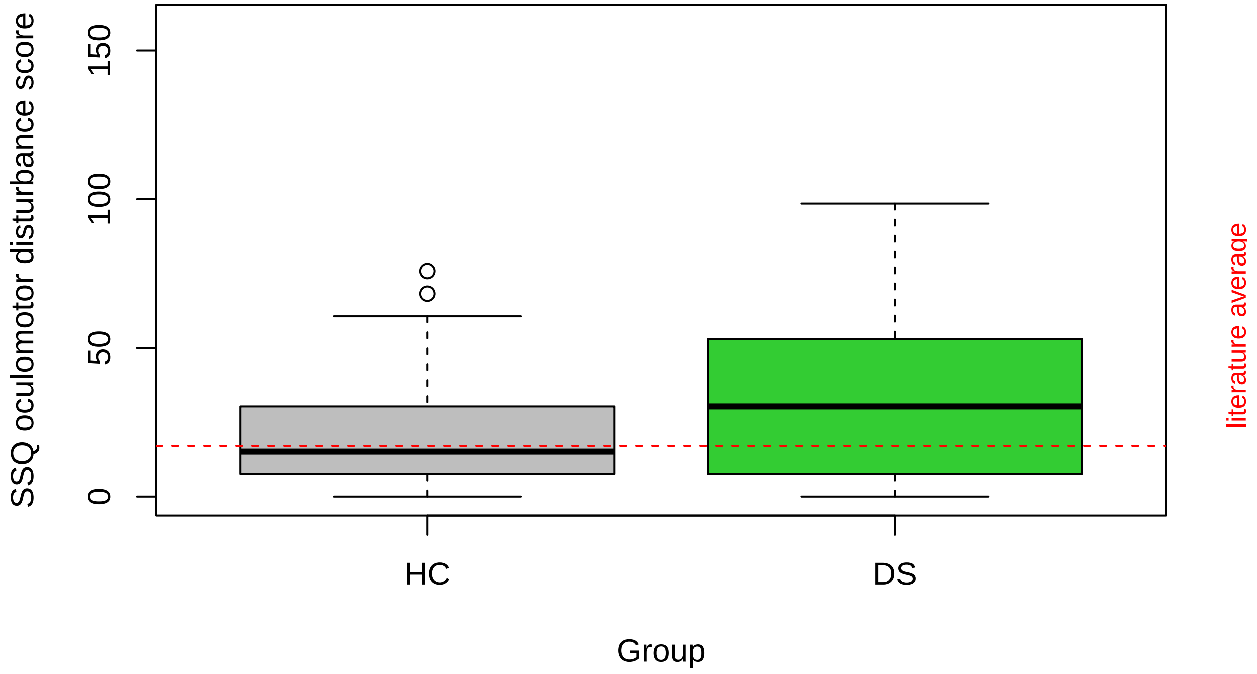


```r
SSQ_boxplotC <- recordPlot()
#print(SSQ_boxplotC)

# Save figure
png("SSQ_Oculomotor_figure.png", units="px", width=1000, height=1800, res=300)
print(SSQ_boxplotC)
dev.off()
```

```
## pdf
##   2
```

```r
## Disorientation
boxplot(d$Disorientation ~ d$Group,
        xlab = "Group",
        ylab = "SSQ disorientation score",
        col = c("grey", "#33cc33"),
        ylim = c(0, 159),            # Set Y-axis limits to the limits of the subscale
        xaxt = 'n')                  # Disable default x-axis)
# Custom x-axis with manually set labels
axis(1,
     at = c(1, 2),  # Positions of the groups on the x-axis
```

```
    labels = c("HC", "DS"))  # Custom labels for 'depressed' and 'control'
# Add horizontal line at literature average
abline(h = 23.50, col = "red", lty = 2) # Literature average based on Saredakis et al., 2020
# Add left-aligned rotated text with custom positioning
text(x = 2.75, y = 28.50, "literature average",
     col = "red", srt = 90, cex = 0.8, xpd = TRUE, adj = c(0, 0))
```

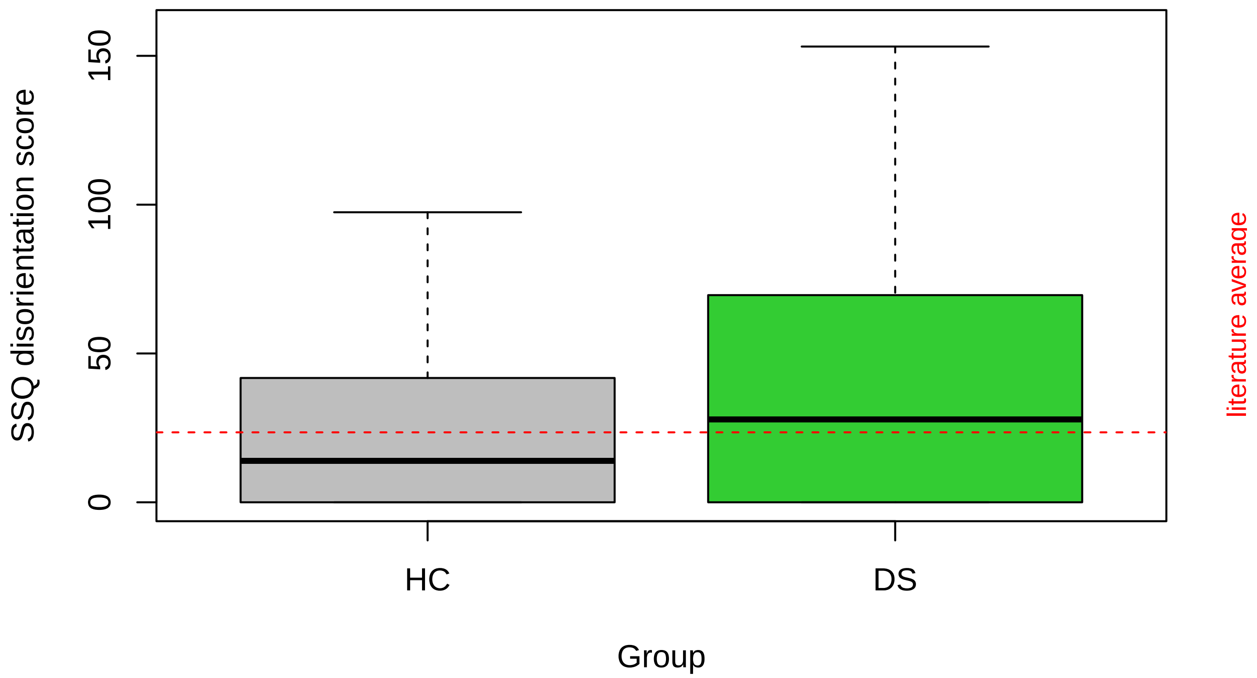


```
SSQ_boxplotD <- recordPlot()
#print(SSQ_boxplotD)

# Save figure
png("SSQ_Disorientation_figure.png", units="px", width=1000, height=1800, res=300)
print(SSQ_boxplotD)
dev.off()
```

```
## pdf
##   2
```

Regression prediction figures

Figure 10. Predicted cybersickness severity levels (with 95% confidence intervals) based on depressive symptom severity, as measured by: (A) the Patient Health Questionnaire and (B) the Depression Anxiety Stress Scales.

```r
## Prediction figure
# Fit the regression model
SSQ_PHQ_pred <- lm(SSQ_Total ~ Age + Education + Gender + PHQ_Tot, data = d)
summary(SSQ_PHQ_pred)
```

```
##
## Call:
## lm(formula = SSQ_Total ~ Age + Education + Gender + PHQ_Tot,
##     data = d)
##
## Residuals:
##     Min      1Q  Median      3Q     Max
## -43.357 -19.226  -5.657  12.997  79.710
##
## Coefficients:
##             Estimate Std. Error t value Pr(>|t|)
## (Intercept)  22.0770    48.5166   0.455 0.650129
## Age          -2.3276     2.2340  -1.042 0.300119
## Education     2.0228     3.3456   0.605 0.546898
## Gender        7.1781     6.9005   1.040 0.300902
## PHQ_Tot       2.2740     0.6354   3.579 0.000548 ***
## ---
## Signif. codes:  0 '***' 0.001 '**' 0.01 '*' 0.05 '.' 0.1 ' ' 1
##
## Residual standard error: 26.5 on 94 degrees of freedom
##   (1 observation deleted due to missingness)
## Multiple R-squared:  0.1426, Adjusted R-squared:  0.1061
## F-statistic: 3.907 on 4 and 94 DF,  p-value: 0.005585
```

```r
confint(SSQ_PHQ_pred)
```

```
##                   2.5 %     97.5 %
## (Intercept) -74.253832 118.407861
## Age          -6.763288   2.107989
## Education    -4.620003   8.665541
## Gender       -6.523021  20.879247
## PHQ_Tot       1.012352   3.535569
```

```r
# Generate predicted values for PHQ_Tot, while holding other predictors constant
predicted_values_PHQ <- ggpredict(SSQ_PHQ_pred, terms = "PHQ_Tot")

# Combine the predicted values with the actual data points for plotting
# Create a dataframe with actual values for plotting
actual_data <- data.frame(
  PHQ_Tot = d$PHQ_Tot,
  SSQ_Total = d$SSQ_Total,
  Group = d$Group  # Include the Group variable
)

# Figure 10.A Plot the predicted values with actual data points and jitter
SSQ_PHQ_plot <- ggplot() +
  # Add the line for predicted values
```

```r
  geom_line(data = predicted_values_PHQ, aes(x = x, y = predicted), color = "blue", size = 1.2) +
  geom_ribbon(data = predicted_values_PHQ, aes(x = x, ymin = conf.low, ymax = conf.high), alpha = 0.2, f
  # Add actual data points with jitter and color by Group
  geom_point(data = actual_data, aes(x = PHQ_Tot, y = SSQ_Total, color = Group), alpha = 0.8, position =
  # Customize the color scale
  scale_color_manual(
    values = c("Control" = "grey", "Depressed" = "#33cc33"),
    labels = c("Healthy control", "Depressive symptoms")  # Customize group names
  ) +
  # Adjust labels and theme
  labs(
    x = "Depression severity (PHQ-9 score)",
    y = "Cybersickness severity (SSQ score)",
    color = "Group"  # Customize legend title
  ) +
  theme_minimal(base_size = 16) +  # Base font size for all text elements
  theme(
    plot.title = element_text(hjust = 0.5, size = 14),    # Increase plot title size
    axis.title = element_text(size = 14),                 # Increase axis titles size
    axis.text = element_text(size = 10),                  # Increase axis text size
    legend.position = c(0.2, 0.9),                        # Position legend inside the plot (x, y)
    legend.background = element_rect(fill = "white", color = "white"),     # Add a white background and
    legend.title = element_text(size = 10),               # Make the legend title smaller
    legend.text = element_text(size = 8),                 # Make the legend labels smaller
    legend.key.size = unit(0.4, "cm"),                    # Reduce the size of the legend key (symbols)
    legend.spacing.y = unit(0.1, "cm")                    # Reduce vertical spacing between legend item
  )
```

```
## Warning: Using ‘size‘ aesthetic for lines was deprecated in ggplot2 3.4.0.
## i Please use ‘linewidth‘ instead.
## This warning is displayed once every 8 hours.
## Call ‘lifecycle::last_lifecycle_warnings()‘ to see where this warning was
## generated.
```

```
## Warning: A numeric ‘legend.position‘ argument in ‘theme()‘ was deprecated in ggplot2
## 3.5.0.
## i Please use the ‘legend.position.inside‘ argument of ‘theme()‘ instead.
## This warning is displayed once every 8 hours.
## Call ‘lifecycle::last_lifecycle_warnings()‘ to see where this warning was
## generated.
```

```r
# Display the plot
print(SSQ_PHQ_plot)
```

```
## Warning: Removed 1 row containing missing values or values outside the scale range
## (‘geom_point()‘).
```

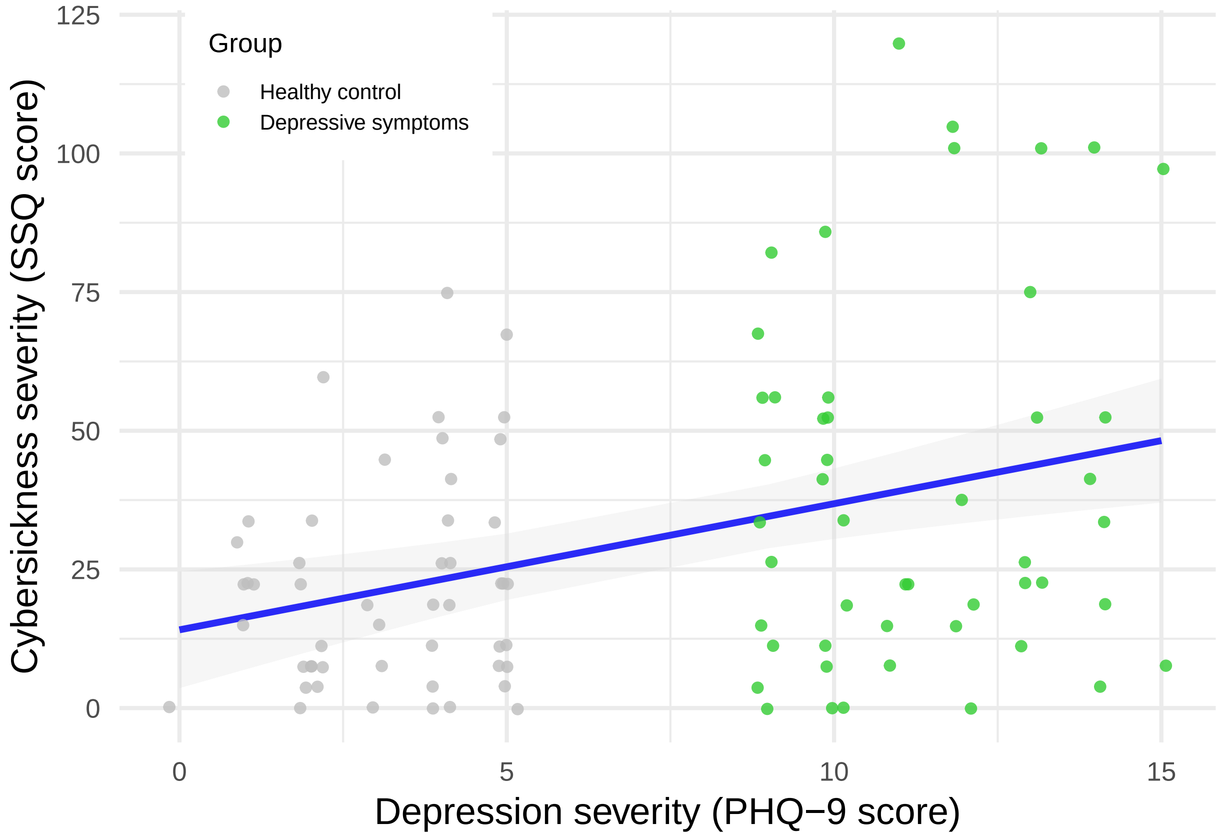


```r
# Save the plot as PNG
png("SSQ_PHQ_pred.png", units = "px", width = 1600, height = 1600, res = 300)
print(SSQ_PHQ_plot)
```

```
## Warning: Removed 1 row containing missing values or values outside the scale range
## (‘geom_point()‘).
```

```r
dev.off()
```

```
## pdf
##   2
```

```r
## Linear prediction figure for DASS-D
# Fit the regression model
SSQ_DASS_pred <- lm(SSQ_Total ~ Age + Education + Gender + DASS_D + DASS_A + DASS_S, data = d)
summary(SSQ_DASS_pred)
```

```
##
## Call:
## lm(formula = SSQ_Total ~ Age + Education + Gender + DASS_D +
##     DASS_A + DASS_S, data = d)
##
## Residuals:
##     Min      1Q  Median      3Q     Max
```

```
## -48.551 -17.213  -5.309  12.065  78.890
##
## Coefficients:
##             Estimate Std. Error t value Pr(>|t|)
## (Intercept)  -7.0753    49.1016  -0.144  0.88574
## Age          -2.9294     2.1790  -1.344  0.18215
## Education     4.4040     3.3787   1.303  0.19567
## Gender       10.8801     6.8411   1.590  0.11518
## DASS_D        1.1491     0.4335   2.651  0.00945 **
## DASS_A        1.2002     0.6375   1.883  0.06291 .
## DASS_S       -0.3181     0.5749  -0.553  0.58140
## ---
## Signif. codes:  0 '***' 0.001 '**' 0.01 '*' 0.05 '.' 0.1 ' ' 1
##
## Residual standard error: 25.73 on 92 degrees of freedom
##   (1 observation deleted due to missingness)
## Multiple R-squared:  0.2089, Adjusted R-squared:  0.1573
## F-statistic: 4.049 on 6 and 92 DF,  p-value: 0.001203
```

```
confint(SSQ_DASS_pred)
```

```
##                     2.5 %     97.5 %
## (Intercept) -104.59532270 90.4448018
## Age           -7.25713408  1.3984110
## Education     -2.30638520 11.1143943
## Gender        -2.70698149 24.4670959
## DASS_D         0.28817410  2.0099729
## DASS_A        -0.06595861  2.4664171
## DASS_S        -1.45999022  0.8237579
```

```
# Generate predicted values for DASS_D, while holding other predictors constant
predicted_values_DASS <- ggpredict(SSQ_DASS_pred, terms = "DASS_D")

# Combine the predicted values with the actual data points for plotting
# Create a dataframe with actual values for plotting
actual_data_DASS <- data.frame(
  DASS_D = d$DASS_D,
  SSQ_Total = d$SSQ_Total,
  Group = d$Group  # Include the Group variable
)

# Figure 10.B. Plot the predicted values with actual data points and jitter
SSQ_DASS_plot <- ggplot() +
  # Add the line for predicted values
  geom_line(data = predicted_values_DASS, aes(x = x, y = predicted), color = "blue", size = 1.2) +
  geom_ribbon(data = predicted_values_DASS, aes(x = x, ymin = conf.low, ymax = conf.high), alpha = 0.2,
  # Add actual data points with jitter and color by Group
  geom_point(data = actual_data_DASS, aes(x = DASS_D, y = SSQ_Total, color = Group), alpha = 0.8, positi
  # Customize the color scale
  scale_color_manual(values = c("Control" = "grey", "Depressed" = "#33cc33")) +
  # Adjust labels and theme
  labs(
    x = "Depression severity (DASS-D score)",
```

```r
    y = "Cybersickness severity (SSQ score)"
  ) +
  theme_minimal(base_size = 16) +  # Base font size for all text elements
  theme(
    plot.title = element_text(hjust = 0.5, size = 14),     # Increase plot title size
    axis.title = element_text(size = 14),                  # Increase axis titles size
    axis.text = element_text(size = 10),                   # Increase axis text size
    legend.position = "none"                              # Remove the legend
  )

# Display the plot
print(SSQ_DASS_plot)
```

```
## Warning: Removed 1 row containing missing values or values outside the scale range
## (`geom_point()`).
```

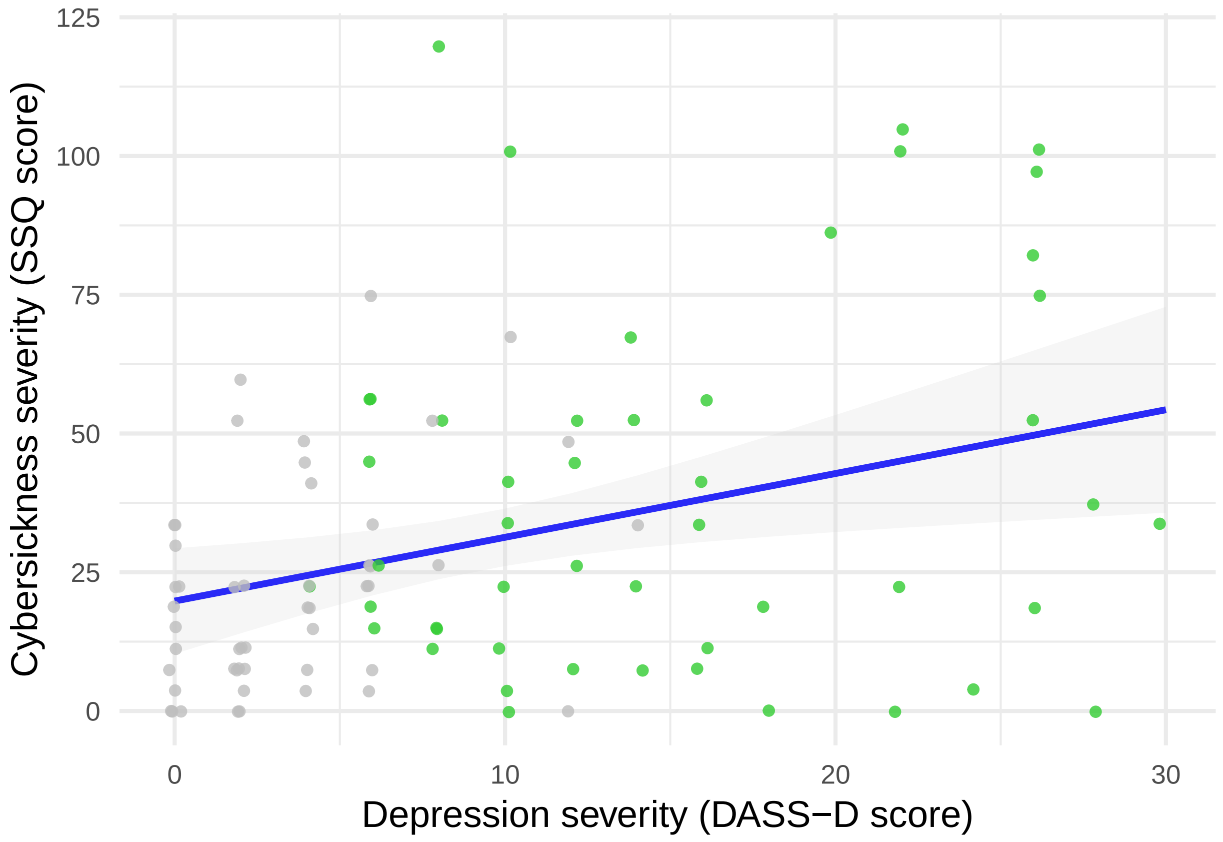


```r
# Save the plot as PNG
png("SSQ_DASS_plot.png", units = "px", width = 1600, height = 1600, res = 300)
print(SSQ_DASS_plot)
```

```
## Warning: Removed 1 row containing missing values or values outside the scale range
## (`geom_point()`).
```

```r
dev.off()
```

```
## pdf
##   2
```

Analyses

Linear regression models

```r
## Total SSQ score
SSQ_demographics <- lm(d$SSQ_Total ~ d$Age + d$Education + d$Gender + d$Group)
summary(SSQ_demographics)
```

```
##
## Call:
## lm(formula = d$SSQ_Total ~ d$Age + d$Education + d$Gender + d$Group)
##
## Residuals:
##     Min      1Q  Median      3Q     Max
## -41.722 -19.680  -4.123  11.075  79.988
##
## Coefficients:
##                   Estimate Std. Error t value Pr(>|t|)
## (Intercept)         37.837     48.272   0.784  0.43511
## d$Age               -2.170      2.261  -0.959  0.33977
## d$Education          1.454      3.370   0.432  0.66707
## d$Gender             5.811      6.965   0.834  0.40627
## d$GroupDepressed    17.584      5.546   3.170  0.00206 **
## ---
## Signif. codes:  0 '***' 0.001 '**' 0.01 '*' 0.05 '.' 0.1 ' ' 1
##
## Residual standard error: 26.85 on 94 degrees of freedom
##   (1 observation deleted due to missingness)
## Multiple R-squared:  0.1199, Adjusted R-squared:  0.0824
## F-statistic:   3.2 on 4 and 94 DF,  p-value: 0.01644
```

```r
confint(SSQ_demographics)
```

```
##                       2.5 %     97.5 %
## (Intercept)      -58.008927 133.683127
## d$Age             -6.659645   2.320205
## d$Education       -5.236955   8.145492
## d$Gender          -8.019250  19.640571
## d$GroupDepressed   6.571965  28.596399
```

```r
mean(d$SSQ_Total[d$Group=="Depressed"], na.rm = T)
```

```
## [1] 39.38449
```

```r
mean(d$SSQ_Total[d$Group=="Control"], na.rm = T)
```

```
## [1] 21.7668
```

```r
sd(d$SSQ_Total[d$Group=="Depressed"], na.rm = T)
```

```
## [1] 32.89023
```

```r
sd(d$SSQ_Total[d$Group=="Control"], na.rm = T)
```

```
## [1] 18.83977
```

```r
## Nause
SSQ_Nausea_demographics <- lm(d$Nausea ~ d$Age + d$Education + d$Gender + d$Group)
summary(SSQ_Nausea_demographics)
```

```
##
## Call:
## lm(formula = d$Nausea ~ d$Age + d$Education + d$Gender + d$Group)
##
## Residuals:
##     Min      1Q  Median      3Q     Max
## -30.720 -14.029  -5.043  13.129  85.122
##
## Coefficients:
##                    Estimate Std. Error t value Pr(>|t|)
## (Intercept)         21.3797    37.6144   0.568  0.57113
## d$Age               -0.5541     1.7621  -0.314  0.75387
## d$Education          0.1002     2.6259   0.038  0.96963
## d$Gender             2.1218     5.4275   0.391  0.69673
## d$GroupDepressed    15.3293     4.3217   3.547  0.00061 ***
## ---
## Signif. codes:  0 '***' 0.001 '**' 0.01 '*' 0.05 '.' 0.1 ' ' 1
##
## Residual standard error: 20.92 on 94 degrees of freedom
##   (1 observation deleted due to missingness)
## Multiple R-squared:  0.1292, Adjusted R-squared:  0.09211
## F-statistic: 3.486 on 4 and 94 DF,  p-value: 0.01063
```

```r
confint(SSQ_Nausea_demographics)
```

```
##                        2.5 %    97.5 %
## (Intercept)      -53.304685 96.064027
## d$Age             -4.052691  2.944514
## d$Education       -5.113640  5.314120
## d$Gender          -8.654663 12.898196
## d$GroupDepressed   6.748445 23.910143
```

```r
mean(d$Nausea[d$Group=="Depressed"], na.rm = T)
```

```
## [1] 29.39878
```

```r
mean(d$Nausea[d$Group=="Control"], na.rm = T)
```

```
## [1] 13.9284
```

```r
## Oculomotor
## Occulomotor
SSQ_Oculomotor_demographics <- lm(d$Oculomotor ~ d$Age + d$Education + d$Gender + d$Group)
summary(SSQ_Oculomotor_demographics)
```

```
##
## Call:
## lm(formula = d$Oculomotor ~ d$Age + d$Education + d$Gender +
##     d$Group)
##
## Residuals:
##     Min      1Q  Median      3Q     Max
## -41.155 -17.343  -3.648  14.346  65.768
##
## Coefficients:
##                   Estimate Std. Error t value Pr(>|t|)
## (Intercept)         54.215     42.713   1.269  0.20747
## d$Age               -3.192      2.001  -1.595  0.11397
## d$Education          2.193      2.982   0.735  0.46389
## d$Gender             2.191      6.163   0.355  0.72302
## d$GroupDepressed    13.509      4.908   2.753  0.00709 **
## ---
## Signif. codes:  0 '***' 0.001 '**' 0.01 '*' 0.05 '.' 0.1 ' ' 1
##
## Residual standard error: 23.76 on 94 degrees of freedom
##   (1 observation deleted due to missingness)
## Multiple R-squared:  0.1075, Adjusted R-squared:  0.06951
## F-statistic:  2.83 on 4 and 94 DF,  p-value: 0.02888
```

```r
confint(SSQ_Oculomotor_demographics)
```

```
##                       2.5 %     97.5 %
## (Intercept)      -30.592580 139.023453
## d$Age             -7.165196   0.780498
## d$Education       -3.727541   8.113729
## d$Gender         -10.046264  14.428141
## d$GroupDepressed   3.764726  23.252738
```

```r
mean(d$Oculomotor[d$Group=="Depressed"], na.rm = T)
```

```
## [1] 33.56857
```

```r
mean(d$Oculomotor[d$Group=="Control"], na.rm = T)
```

```
## [1] 20.0112
```

```r
## Disorientation
SSQ_Disorientation_demographics <- lm(d$Disorientation ~ d$Age + d$Education + d$Gender + d$Group)
summary(SSQ_Disorientation_demographics)
```

```
##
## Call:
## lm(formula = d$Disorientation ~ d$Age + d$Education + d$Gender +
##     d$Group)
##
## Residuals:
##    Min     1Q Median     3Q    Max
## -47.85 -27.84 -12.66  16.02 150.17
##
## Coefficients:
##                  Estimate Std. Error t value Pr(>|t|)
## (Intercept)        10.070     70.246   0.143   0.8863
## d$Age              -1.405      3.291  -0.427   0.6705
## d$Education         1.239      4.904   0.253   0.8011
## d$Gender           14.507     10.136   1.431   0.1557
## d$GroupDepressed   18.272      8.071   2.264   0.0259 *
## ---
## Signif. codes:  0 '***' 0.001 '**' 0.01 '*' 0.05 '.' 0.1 ' ' 1
##
## Residual standard error: 39.07 on 94 degrees of freedom
##   (1 observation deleted due to missingness)
## Multiple R-squared:  0.07758,    Adjusted R-squared:  0.03832
## F-statistic: 1.976 on 4 and 94 DF,  p-value: 0.1044
```

```r
confint(SSQ_Disorientation_demographics)
```

```
##                        2.5 %     97.5 %
## (Intercept)      -129.404987 149.543991
## d$Age              -7.938295   5.129121
## d$Education        -8.498029  10.976016
## d$Gender           -5.617724  34.632658
## d$GroupDepressed    2.247242  34.297048
```

```r
mean(d$Disorientation[d$Group=="Depressed"], na.rm = T)
```

```
## [1] 42.04408
```

```r
mean(d$Disorientation[d$Group=="Control"], na.rm = T)
```

```
## [1] 23.9424
```

Compared to literature average

```r
## Check for statistical significance compared to literature average (Saredakis et al., 2020)
# Define variable of interest and literature average to compare to
# SSQ_Total - 28
# Nausea - 16.72
# Oculomotor - 17.09
# Disorientation - 23.50
d$interest   <- d$SSQ_Total
literature <- 28

# Shapiro-Wilk test on the full sample
shapiro_full <- shapiro.test(d$interest)
print(shapiro_full)
```

```
##
##  Shapiro-Wilk normality test
##
## data:  d$interest
## W = 0.87367, p-value = 1.109e-07
```

```r
# Wilcoxon signed rank test on the full sample
wilcox_full <- wilcox.test(d$interest, mu = literature)
print(wilcox_full)
```

```
##
##  Wilcoxon signed rank test with continuity correction
##
## data:  d$interest
## V = 2450, p-value = 0.9318
## alternative hypothesis: true location is not equal to 28
```

```r
# Calculate effect size
effect_size_w <- rank_biserial(
  x = d$interest,
  mu = literature,
  alternative = "two.sided"
)
```

```
## Warning: Missing values detected. NAs dropped.
```

```r
print(effect_size_w)
```

```
## r (rank biserial) |        95% CI
## ---------------------------------
## -0.01             | [-0.23, 0.21]
##
## - Deviation from a difference of 28.
```

```r
# Descriptives
mean(d$interest, na.rm = T)
```

```
## [1] 30.48667
```

```r
sd(d$interest, na.rm = T)
```

```
## [1] 28.03022
```

```r
# Subset data
depressed_group <- subset(d, Group == "Depressed")
control_group <- subset(d, Group == "Control")

# Shapiro-Wilk test
shapiro_depressed <- shapiro.test(depressed_group$interest)
print(shapiro_depressed)
```

```
##
##  Shapiro-Wilk normality test
##
## data:  depressed_group$interest
## W = 0.90378, p-value = 0.0007315
```

```r
shapiro_control <- shapiro.test(control_group$interest)
print(shapiro_control)
```

```
##
##  Shapiro-Wilk normality test
##
## data:  control_group$interest
## W = 0.90419, p-value = 0.0006626
```

```r
## Wilcoxon Signed-Rank test for subgroups
wilcox_depressed <- wilcox.test(depressed_group$interest, mu = literature)
```

```
## Warning in wilcox.test.default(depressed_group$interest, mu = literature):
## cannot compute exact p-value with ties
```

```r
print(wilcox_depressed)
```

```
##
##  Wilcoxon signed rank test with continuity correction
##
## data:  depressed_group$interest
## V = 798, p-value = 0.06564
## alternative hypothesis: true location is not equal to 28
```

```r
# Calculate effect size
effect_size_w <- rank_biserial(
  x = depressed_group$interest,
  mu = literature,
  alternative = "two.sided"
)
```

```
## Warning: Missing values detected. NAs dropped.
```

```r
print(effect_size_w)
```

```
## r (rank biserial) |        95% CI
## ---------------------------------
## 0.30              | [-0.01, 0.56]
##
## - Deviation from a difference of 28.
```

```r
wilcox_control <- wilcox.test(control_group$interest, mu = literature)
print(wilcox_control)
```

```
##
##  Wilcoxon signed rank test with continuity correction
##
## data:  control_group$interest
## V = 402, p-value = 0.02315
## alternative hypothesis: true location is not equal to 28
```

```r
# Calculate effect size
effect_size_w <- rank_biserial(
  x = control_group$interest,
  mu = literature,
  alternative = "two.sided"
)
print(effect_size_w)
```

```
## r (rank biserial) |         95% CI
## ----------------------------------
## -0.37             | [-0.61, -0.07]
##
## - Deviation from a difference of 28.
```

```r
mean(depressed_group$Oculomotor, na.rm = T)
```

```
## [1] 33.56857
```

```r
sd(depressed_group$Oculomotor, na.rm = T)
```

```
## [1] 27.50471
```

Request session information

```
sessionInfo()
```

```
## R version 4.4.0 (2024-04-24)
## Platform: aarch64-apple-darwin20
## Running under: macOS Sonoma 14.7.4
##
## Matrix products: default
## BLAS:   /Library/Frameworks/R.framework/Versions/4.4-arm64/Resources/lib/libRblas.0.dylib
## LAPACK: /Library/Frameworks/R.framework/Versions/4.4-arm64/Resources/lib/libRlapack.dylib;  LAPACK ve
##
## locale:
## [1] en_US.UTF-8/en_US.UTF-8/en_US.UTF-8/C/en_US.UTF-8/en_US.UTF-8
##
## time zone: Europe/Stockholm
## tzcode source: internal
##
## attached base packages:
## [1] splines   stats4    stats     graphics  grDevices utils     datasets
## [8] methods   base
##
## other attached packages:
##  [1] effectsize_1.0.0  rmarkdown_2.28    VGAM_1.1-12       ggeffects_1.7.1
##  [5] coin_1.4-3        survival_3.5-8    rstatix_0.7.2     psych_2.4.6.26
##  [9] MASS_7.3-61       brant_0.3-0       ordinal_2023.12-4 vioplot_0.4.0
## [13] zoo_1.8-12        sm_2.2-6.0        readxl_1.4.3      lubridate_1.9.3
## [17] forcats_1.0.0     stringr_1.5.1     purrr_1.0.2       readr_2.1.5
## [21] tidyr_1.3.1       tibble_3.2.1      ggplot2_3.5.1     tidyverse_2.0.0
## [25] dplyr_1.1.4
##
## loaded via a namespace (and not attached):
##  [1] tidyselect_1.2.1    libcoin_1.0-10      farver_2.1.2
##  [4] fastmap_1.2.0       TH.data_1.1-2       bayestestR_0.15.0
##  [7] digest_0.6.35       timechange_0.3.0    lifecycle_1.0.4
## [10] magrittr_2.0.3      compiler_4.4.0      rlang_1.1.4
## [13] tools_4.4.0         utf8_1.2.4          yaml_2.3.8
## [16] knitr_1.47          labeling_0.4.3      mnormt_2.1.1
## [19] abind_1.4-5         multcomp_1.4-25     withr_3.0.0
## [22] numDeriv_2016.8-1.1 grid_4.4.0          datawizard_1.0.0
## [25] fansi_1.0.6         colorspace_2.1-0    scales_1.3.0
## [28] insight_1.0.1       cli_3.6.2           mvtnorm_1.2-5
## [31] crayon_1.5.2        generics_0.1.3      rstudioapi_0.16.0
## [34] tzdb_0.4.0          parameters_0.24.1   modeltools_0.2-23
## [37] parallel_4.4.0      cellranger_1.1.0    matrixStats_1.3.0
## [40] vctrs_0.6.5         boot_1.3-30         Matrix_1.7-0
## [43] sandwich_3.1-0      carData_3.0-5       car_3.1-2
## [46] hms_1.1.3           glue_1.7.0          codetools_0.2-20
## [49] stringi_1.8.4       gtable_0.3.5        munsell_0.5.1
## [52] pillar_1.9.0        htmltools_0.5.8.1   R6_2.5.1
## [55] ucminf_1.2.2        evaluate_0.24.0     lattice_0.22-6
## [58] haven_2.5.4         highr_0.11          backports_1.5.0
```

```
## [61] broom_1.0.6         nlme_3.1-164        xfun_0.44
## [64] pkgconfig_2.0.3
```
